# Global Impacts Dataset of Invasive Alien Species (GIDIAS)

**DOI:** 10.1038/s41597-025-05184-5

**Published:** 2025-05-21

**Authors:** Sven Bacher, Ellen Ryan-Colton, Mario Coiro, Phillip Cassey, Bella S. Galil, Martin A. Nuñez, Michael Ansong, Katharina Dehnen-Schmutz, Georgi Fayvush, Romina D. Fernandez, Ankila J. Hiremath, Makihiko Ikegami, Angeliki F. Martinou, Shana M. McDermott, Cristina Preda, Montserrat Vilà, Olaf L. F. Weyl, Neelavar Ananthram Aravind, Ioanna Angelidou, Katerina Athanasiou, Vidyadhar Atkore, Jacob N. Barney, Tim M. Blackburn, Eckehard G. Brockerhoff, Clinton Carbutt, Luca Carisio, Pilar Castro-Díez, Vanessa Céspedes, Aikaterini Christopoulou, Diego F. Cisneros-Heredia, Meghan Cooling, Maarten de Groot, Jakovos Demetriou, James W. E. Dickey, Virginia G. Duboscq-Carra, Regan Early, Thomas G. Evans, Paola T. Flores-Males, Belinda Gallardo, Monica Gruber, Cang Hui, Jonathan M. Jeschke, Natalia Z. Joelson, Mohd Asgar Khan, Sabrina Kumschick, Lori Lach, Katharina Lapin, Simone Lioy, Chunlong Liu, Zoe J. MacMullen, Manuela A. Mazzitelli, John Measey, Agata A. Mrugała-Koese, Camille L. Musseau, Helen F. Nahrung, Alessia Pepori, Luis R. Pertierra, Elizabeth F. Pienaar, Petr Pyšek, Gonzalo Rivas Torres, Henry A. Rojas Martinez, Julissa Rojas-Sandoval, Ned L. Ryan-Schofield, Rocío M. Sánchez, Alberto Santini, Davide Santoro, Riccardo Scalera, Lisanna Schmidt, Tinyiko Cavin Shivambu, Sima Sohrabi, Elena Tricarico, Alejandro Trillo, Pieter van’t Hof, Lara Volery, Tsungai A. Zengeya

**Affiliations:** 1 Department of Biology, University of Fribourg, Fribourg, Switzerland; 2 Research Institute for the Environment and Livelihoods, Charles Darwin University, Alice Springs, Australia; 3 Senckenberg Research Institute and Natural History Museum, Frankfurt am Main, Germany; 4 School of Biological Sciences, University of Adelaide, Adelaide, SA 5000 Australia; 5 Steinhardt Museum of Natural History and Israel National Center for Biodiversity Studies, Tel Aviv University, Tel Aviv, Israel; 6 Department of Biology and Biochemistry, University of Houston, Houston, TX USA; 7 Department of Silviculture and Forest Management, Kwame Nkrumah University of Science and Technology, Kumasi, Ghana; 8 Centre for Agroecology, Water and Resilience, Coventry University, Ryton Gardens, Coventry, CV8 3LG UK; 9 Institute of Botany after A. Takhtajyan NAS RA, Yerevan, Armenia; 10 Instituto de Ecología Regional, Universidad Nacional de Tucumán-CONICET, Yerba Buena, Tucumán, Argentina; 11 Ashoka Trust for Research in Ecology and the Environment (ATREE), Srirampura, Jakkur Post, Bangalore, 560064 India; 12 Lake Biwa Branch Office, National Institute for Environmental Studies, Otsu, Shiga Japan; 13 Climate and Atmosphere Research Center (CARE-C), The Cyprus Institute, Nicosia, Cyprus; 14 Department of Economics, University of Missouri, Columbia, MO USA; 15 Department of Natural Sciences, Ovidius University of Constanta, Constanta, Romania; 16 Departamento de Biología Vegetal y Ecología, Universidad de Sevilla, 41092 Sevilla, Spain; 17 Estación Biológica de Doñana, EBD-CSIC, Sevilla, Spain; 18 South African Institute for Aquatic Biodiversity, Grahamstown, South Africa; 19 Joint Services Health Unit, British Forces, Cyprus; 20 Salim Ali Centre for Ornithology and Natural History (SACON), Coimbatore, India; 21 Virginia Tech /, Blacksburg, VA USA; 22 Centre for Biodiversity and Environment Research, Department of Genetics, Evolution and Environment, University College London, London, UK; 23 Institute of Zoology, Zoological Society of London, London, UK; 24 Swiss Federal Research Institute WSL, Birmensdorf, Switzerland; 25 School of Life Sciences, University of KwaZulu-Natal, Scottsville, 3209 South Africa; 26 Istituto Zooprofilattico Sperimentale del Piemonte Liguria e Valle d’Aosta, Torino, Italy; 27 Department of Life Sciences, University of Alcalá, Alcalá, Spain; 28 Ecology Aquatic and Microscopy Laboratory, Estación Biológica de Doñana, EBD-CSIC, Sevilla, Spain; 29 Department of Ecology and Systematics, Faculty of Biology, National and Kapodistrian University of Athens, Athens, Greece; 30 Colegio de Ciencias Biológicas y Ambientales, Universidad San Francisco de Quito (USFQ), Quito, Ecuador; 31 Pacific Biosecurity / Victoria University of Wellington, Wellington, New Zealand; 32 Slovenian Forestry Institute, Ljubljana, Slovenia; 33 Leibniz-Institute of Freshwater Ecology and Inland Fisheries (IGB), Berlin, Germany; 34 Freie Universität Berlin, Berlin, Germany; 35 GEOMAR Helmholtz Centre for Ocean Research, Kiel, Germany; 36 CONICET Patagonia Norte, Universidad Nacional de la Plata, La Plata, Argentina; 37 Centre for Ecology and Conservation, University of Exeter, Penryn Campus, Cornwall, UK; 38 Instituto Pirenaico de Ecologia (IPE), Spanish National Research Council (CSIC), Zaragoza, Spain; 39 Te Herenga Waka—Victoria University of Wellington, Wellington, New Zealand; 40 Centre for Invasion Biology, Department of Mathematical Sciences, Stellenbosch University, Stellenbosch, South Africa; 41 Georg-August University of Goettingen, Goettingen, Germany; 42 Department of Botany, University of Kashmir, Srinagar, India; 43 Centre for Invasion Biology, Department of Botany and Zoology, Stellenbosch University, Stellenbosch, South Africa; 44 South African National Biodiversity Institute, Kirstenbosch Research Centre, Kirstenbosch, South Africa; 45 Centre for Tropical Biosecurity, James Cook University, Cairns, Australia; 46 Austrian Research Centre for Forests, Vienna, Austria; 47 Department of Agricultural, Forest and Food Sciences, University of Turin, Grugliasco, Italy; 48 Key Laboratory of Mariculture, Ministry of Education, College of Fisheries, Ocean University of China, Qingdao, China; 49 ICPA, Universidad de Tierra del Fuego, Ushuaia, Argentina; 50 Institute of Biodiversity, Yunnan University, Kunming, China; 51 Forest Research Institute / University of the Sunshine Coast, Brisbane, Australia; 52 Institute for Sustainable Plant Protection - C.N.R, Sesto Fiorentino, Italy; 53 National Museum of Natural Sciences (CSIC-MNCN), Madrid, Spain; 54 Warnell School of Forestry and Natural Resources, University of Georgia, 180 E. Green Street, Athens, Georgia USA; 55 Institute of Botany, Czech Academy of Sciences, Průhonice, Czech Republic; 56 Estación de Biodiversidad Tiputini, Universidad San Francisco de Quito, Quito, Ecuador; 57 Institute of the Environment & Department of Geography, Sustainability, Community, and Urban Studies, University of Connecticut, Storrs, Connecticut USA; 58 Instituto de Diversidad y Ecología Animal, Universidad Nacional de Córdoba-CONICET, Córdoba, Argentina; 59 National Research Council, Institute for Sustainable Plant Protection, Sesto fiorentino, Italy; 60 Ministry for Primary Industries, Biosecurity New Zealand /, Wellington, New Zealand; 61 IUCN/SSC Invasive Species Specialist Group, Rome, Italy; 62 Estonian Academy of Arts, Tallinn, Estonia; 63 Department of Environmental Sciences, University of South Africa, Roodepoort, South Africa; 64 Ferdowsi University of Mashhad, Gorgan, Iran; 65 Department of Biology, University of Florence, Sesto Fiorentino, FI Italy; 66 Centre for Invasion Biology, Department of Zoology and Entomology, University of Pretoria, Pretoria, South Africa

**Keywords:** Invasive species, Interdisciplinary studies

## Abstract

Invasive alien species are a major driver of global change, impacting biodiversity, ecosystem services, and human livelihoods. To document these impacts, we present the Global Impacts Dataset of Invasive Alien Species (GIDIAS), a dataset on the positive, negative and neutral impacts of invasive alien species on nature, nature’s contributions to people, and good quality of life. This dataset arises from the Intergovernmental Science-Policy Platform on Biodiversity and Ecosystem Services’ (IPBES) thematic assessment report of this topic. Data were compiled from published sources, including grey literature, reporting a direct observation of an invasive alien species’ impact. All impact records contain up to 52 fields of contextual information and attempt to link impacts to the global standard “environmental impact classification for alien taxa” (EICAT) and “socio-economic impact classification for alien taxa” (SEICAT). GIDIAS includes more than 22000 records of impacts caused by 3353 invasive alien species (plants, vertebrates, invertebrates, microorganisms) from all continents and realms (terrestrial, freshwater, marine), extracted from over 6700 sources. We intend GIDIAS to be a global resource for investigating and managing the variety of impacts of invasive alien species across taxa and regions.

## Background & Summary

Invasive alien species (IAS) are defined by the International Union for Conservation of Nature (IUCN) as species introduced by humans to places outside their natural range and that have negative impacts on native biodiversity, ecosystem services or human economy and well-being^1^. Not all alien species have documented negative impacts and thus are considered as invasive; some alien species have impacts that can be positive for some native species or for people. Globally, about 40,000 species from all taxonomic groups and realms have known alien populations^2^, a trend predicted to increase by 36% globally over the next 30 years^3^. Introduction rates of alien species to regions in which they never previously occurred increased steadily over the last centuries and have now reached an unprecedented yearly global rate of approximately 200 newly documented alien species^4^. About 25% of the first records of alien species in a given country or territory consist of species that have never been previously documented as alien in any part of the world^5^, underscoring the difficulty of predicting and mitigating their impacts when there is no prior data or global experience to guide management efforts.

It remains crucial to investigate, understand, and, wherever feasible, anticipate the impacts that these species have on recipient ecosystems and society^6^. This task is particularly challenging due to the complex and multifaceted nature of these impacts, which can vary largely across taxonomic, ecological, and societal contexts. Here, we use the Intergovernmental Science-Policy Platform on Biodiversity and Ecosystem Services (IPBES) conceptual framework as a model of interactions between nature and people^7,8^ to address the impacts of invasive alien species. One approach to classify impacts is by considering how biological invasions modify nature, nature’s contributions to people, and good quality of life^9^. Impacts might range along a continuum from nearly indiscernible to large and widespread changes. While some impacts might be detrimental to native biodiversity or people (negative impacts), others might be beneficial (positive impacts^10,11^). Appreciation of the extent, direction, and intensity of impacts is essential for prioritising appropriate policy and governance responses to biological invasions.

Recognizing this need, we present the Global Impacts Dataset of Invasive Alien Species (GIDIAS), a global dataset of 22865 records including impacts of invasive alien species on nature, nature’s contributions to people, and good quality of life (see below for definitions). Records include positive and negative impacts, neutral impacts (studies were carried out, but no impacts were documented), non-directional impacts (i.e., change without detriments or benefits for native species or people), and finally, some records of alien species where no studies were found that assessed their impacts (indicating data gaps). Records cover 3353 invasive alien species from all major taxa (plants, vertebrates, invertebrates, microorganisms) and all continents and realms (terrestrial, freshwater, marine). The data were compiled to serve as robust evidence for the global assessment report on invasive alien species by the Intergovernmental Science-Policy Platform on Biodiversity and Ecosystem Services^2,9^.

### Types of impact

The impacts of alien species are generally defined as changes to an ecosystem (i.e., impacts to nature) or socio-economic system (i.e., nature’s contribution to people and good quality of life). Impacts to nature, or ‘ecological impacts’, are defined as measurable changes to the ecological properties of the recipient ecosystem^12^. This implies that all alien species have the potential to cause impacts, even when not yet forming established populations or being widespread over larger areas, simply by their presence and integration into the ecosystem. These changes can occur at all levels of ecological complexity. Thus, impacts can be measured at the level of an organism (e.g., changes in survival, growth, reproduction), a population (e.g., changes in abundance, density), a community (e.g., changes in species richness, evenness, composition, trophic structure), or an ecosystem (e.g., changes in physical, chemical and structural habitat properties, nutrient and water cycling, decomposition rates, energy flow).

Nature’s contribution to people (NCP) is a framework that builds on the ecosystem service concept but places ecosystem services within the context of broad and diverse socio-cultural connections between people and nature^8^, such that nature makes contributions to people through material, regulating and non-material ways. IPBES describes 18 categories of nature’s contribution to people^8^, which range from the provision of materials, including energy, food and medicines, through to regulation services such as pollination, habitat creation or regulation of water quality, and finally non-material contributions such as opportunities for learning or supporting identities (Table 1). Invasive alien species may alter these contributions to people in positive or negative ways. Impacts to nature’s contributions to people can be positive, e.g., increase in food availability (NCP 12), protection from erosion (NCP 8), as well as negative, e.g., exacerbating fire hazards (NCP 9), soil erosion (NCP 8), allergenic pollen, zoonotic diseases, poisoning and envenomation (all NCP 10)^13^.

**Table 1:** Nature’s contributions to people (NCP) and their contribution to material, non-material and regulating services (after Diaz *et al*.^8^).

Nature’s contributions to people	Material	Non-material	Regulating
NCP 1: Habitat creation and maintenance			X
NCP 2: Pollination and dispersal of seeds and other propagules			X
NCP 3: Regulation of air quality			X
NCP 4: Regulation of climate			X
NCP 5: Regulation of ocean NCP acidification			X
NCP 6: Regulation of freshwater quantity, location and timing			X
NCP 7: Regulation of freshwater and coastal water quality			X
NCP 8: Formation, protection and decontamination of soils and sediments			X
NCP 9: Regulation of hazards and extreme events			X
NCP 10: Regulation of detrimental organisms and biological processes			X
NCP 11: Energy	X	(X)	
NCP 12: Food and feed	X	(X)	
NCP 13: Materials, companionship and labour	X	(X)	
NCP 14: Medicinal, biochemical and genetic resources	X	(X)	
NCP 15: Learning and inspiration	(X)	X	
NCP 16: Physical and psychological experiences	(X)	X	
NCP 17: Supporting identities	(X)	X	
NCP 18: Maintenance of options	X	X	X

Good quality of life refers to the determinants of human well-being^14^. Good quality of life has different constituents, which can be classified as material and immaterial assets for people (e.g., the provisioning of food and energy; security; health; good social relations; and freedom of choice and action). Each constituent of good quality of life is vulnerable to alteration by invasive alien species, positively and negatively, which can affect peoples’ lives and their preferred activities^15,16^. In GIDIAS, impacts on good quality of life are assessed and measured by observed changes in people’s health and activities rather than changes in environmental properties that could be inferred to pose a health risk, for example. Our approach to assessing impacts on good quality of life is adopted from the Socio-Economic Impact Classification for Alien Taxa (SEICAT) approach^15^.

The dataset distinguishes between impacts on nature, nature’s contributions to people, and good quality of life: impacts on nature refers to impacts on native species, communities, and ecosystem properties; impacts to nature’s contribution to people refers to the change in ecosystem contributions to people, measured in changes to environmental or social parameters, but not how people are consequently affected; and impacts to good quality of life are measured as changes to people’s activities and health. As is apparent, there may be connections between impacts to nature, nature’s contribution to people, and good quality of life, and this dataset might provide a foundation for exploring the more complex impacts of invasive alien species across types rather than in isolation.

### Directionality of Impacts

Impact directionality (i.e., whether impacts of invasive alien species are assessed as ‘negative’ or ‘positive’) is partly grounded in subjective perceptions embedded within economic, cultural, and social contexts^11^. Perception of impacts as positive or negative depends on value systems, and values can vary across or even within the same economic, cultural, and social contexts^17^. For instance, the perception of whether nature or its elements are harmed or benefited can differ significantly among people. Therefore, a clear definition of the entities being evaluated (e.g., native species, people) and the metrics used to assess impact direction and magnitude are crucial for accurately interpreting the changes caused by alien species^11^. To provide clarity amidst these varying perceptions, this dataset defines the impacts of alien species on nature as negative when a native species suffers disadvantage, following the Environmental Impact Classification for Alien Taxa (EICAT) first designed by Blackburn *et al*.^18^ and later endorsed as global standard by the IUCN^19^. Impacts on nature are defined as positive when a native species benefits from ecosystem changes due to the introduction of an invasive alien species^20^. Details on EICAT classification, including positive impacts, are given in Supplementary Materials 1. Note that not all ecosystem changes caused by invasive alien species can be assigned a clear directionality. For example, abiotic characteristics of ecosystems (e.g., changes in soil or water chemistry, structural complexity) can increase or decrease due to the impacts of an invasive alien species, but assigning a direction (positive or negative) is not always straightforward. This is because these changes might have varying consequences for different native species. Moreover, the same abiotic ecosystem change can sometimes be quantified as either an increase or a decrease of an indicator (e.g., an increase in the concentration of hydrogen ions (H+) is equivalent to a decrease in the pH value). To address this complexity, impacts describing abiotic changes in ecosystem characteristics are classified as negative or positive only when there is documented evidence of their consequences, specifically whether they harm or benefit native species. However, when the consequences of such abiotic changes were studied but cannot be linked to a measurable benefit or harm to native species, the impacts are classified as ‘neutral’. By including neutral impacts, the dataset acknowledges impacts where the directionality of change remains ambiguous, ensuring a more comprehensive representation of the diverse effects invasive alien species can have on ecosystems.

Biological invasions can provide benefits or harm to people, which determines the directionality of impacts on nature’s contributions to people and good quality of life. Directional changes in nature’s contributions to people, i.e., increases or decreases of the measured parameters, may be positive or negative for people. For this dataset, positive impacts on nature’s contributions to people are documented as an increase in services or a decrease in disservices, whereas negative impacts would do the opposite^13^. By contrast, for this dataset, directionality in good quality of life is assessed according to SEICAT by changes in peoples’ activities or health through constituents of human well-being^15^. Positive impacts are assigned when people benefit from alien species, and negative when people are harmed. Details on SEICAT classification are given in Supplementary Materials 1.

### Context-dependency of impacts: including additional variables

In addition to the core aim of collating data on multiple types of impacts and their directionality, we aimed to achieve global coverage of impacts across all continents, major ecosystem realms (terrestrial, freshwater, marine), and all major taxonomic groups of invasive alien species (plants, vertebrates, invertebrates, microorganisms). Within this framework, we aimed to record related attributes that would facilitate addressing emerging questions of biological invasions and allow the exploration and testing of the context dependency of impacts. Thus, where available, we documented for each impact record information about the impact location (e.g., whether the location was on an island or in a protected area, the habitat types) and the native species affected. We also collected bibliometric and methodological data, e.g., on the source language, source type (e.g., peer-reviewed, grey literature), and study type (e.g., observational, experimental, field-based, laboratory). These data are valuable for analyses on data coverage and gaps. Certain fields were standardised across the dataset, while others - such as the original text from the source document - were retained as free text, enabling end users to refine and explore the data further for their specific objectives (Supplementary Materials 2).

## Methods

**Fig. 1:**
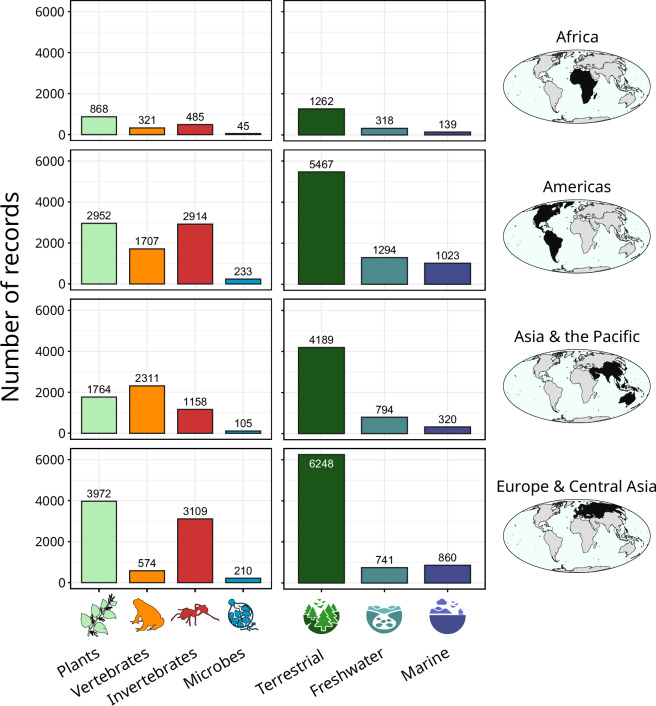
Coverage of impact records in GIASID for taxa and realms across regions.

Protocols for search strategies and a standardised data recording template were developed during an in-person meeting of assessors in August 2019 before data collection began, which was used amongst all assessors. Continuous standardisation of the search strategy and recording template across assessors occurred during eleven additional online meetings between October 2019 and September 2021. Data searches were conducted by 114 assessors working in collaborative groups, conducting searches in 16 languages, resulting in 37 separate datasets (Supplementary Materials 3). Data standardisation and removal of duplicates (described below) led to 22865 impact records in the final, merged dataset (Fig. 1).

### Detailed description of the data gathering

Literature searches were conducted at the global level for impacts of all invasive alien taxa in the marine realm, and globally for specific invasive alien taxonomic groups where global reviews existed (e.g. birds^21,22^; ants^23^; amphibians^24^; ungulates^25^). In addition, literature searches were carried out separately for different regions of the world covering multiple invasive alien taxa in the freshwater and terrestrial realms. Broad regions were defined as Americas, Asia Pacific, Europe and Central Asia, and Africa (Fig. 1), and within each region many subregions were defined and covered, including searches in multiple languages. In each region, specific taxa were assigned to different authors (based on their expertise in that region and taxon), thus generating literature searches by region/taxa combinations (Supplementary Materials 3). All literature searches were conducted between September 2019 and April 2021.

Four main search strategies were used within each region/taxon combination: *checklist*, *broad, snowballing* and/or use of *existing reviews* (the specific search strategies for each region/taxon combination are detailed in Supplementary Materials 3). All searches followed the EICAT search protocol from the IUCN^19^, which is generic enough to capture impacts on nature, nature’s contributions to people, and good quality of life. Some flexibility was permitted for each search guided by the assessor’s expertise in the specific region/taxa combination (Supplementary Materials 3). Searches were conducted across a range of search engines (e.g., Web of Science WOS; Google Scholar; Scopus) that primarily covered scientific published literature (peer-reviewed) but through snowballing (see below) and addition of general search engines (e.g., Google) led to the inclusion of other source types (grey literature).

For some region/taxa combinations, searches started with a *checklist* of alien species in a region. For each invasive alien species on the list and each region, the scientific and common name(s) of the focal invasive alien species was searched in combination with standardised search strings of “alien” and its most common synonyms (e.g., non-native, exotic, non-indigenous, invasive). Regional lists of invasive alien species included country lists, e.g., from the Global Register of Introduced and Invasive Species (GRIIS^26^), European Alien Species Information Network (EASIN^27^) or the National Invasive Species Information Center (NISIC, https://www.invasivespeciesinfo.gov) for the United States of America. Other searches used a *broad* strategy, whereby standardised terms as above were used in combination with “impact” and defined taxonomic groups or local regions rather than searching for each alien taxon individually. Broad searches included additional terms for impacts on nature’s contributions to people or good quality of life (e.g. “food”, “health”). References cited within the sources revealed by the first two search strategies were also included if relevant. This procedure is known as *snowballing* and included a range of sources not restricted to peer-reviewed literature, including theses, government reports, videos, and indigenous and local knowledge sources (source types were recorded in the dataset). Finally, some *existing reviews* found during the searches contained compiled datasets of references to primary sources of observed impacts of specific alien taxa or regions (e.g.^28,29^). Only sources containing direct observations of impacts were selected; potential, extrapolated, or modelled impacts were not recorded. Note that the same source can have descriptions of multiple impact records.

Each impact record refers to an invasive alien species in peer-reviewed or grey literature that a) causes at least one type of impact on nature, nature’s contributions to people, or good quality of life at a location and point in time, or b) had direct observations testing for impact but no impact was found (neutral or non-directional - often the case with measurements of ecosystem parameters), or c) where a checklist search strategy was used, and the species was documented as an alien species but no impact was found in the literature. We are aware that records of neutral impacts are not comprehensive to the same extent for all region/taxa combinations in our dataset because search strategies were not specifically looking for studies of neutral impacts. Thus, care must be taken in the interpretation and use of neutral impacts in statistical analyses. However, we included them because we foresee/anticipate that neutral impacts will be modified in future updates of this dataset and to highlight current knowledge gaps.

### Impact magnitudes

For each impact record, we assigned impact magnitudes to nature, nature’s contributions to people, and good quality of life, if applicable. Magnitudes for negative impacts on nature follow the EICAT system from the IUCN^19^ in four levels, namely: no impact found (level 0); impacts on performance of native individuals (level 1 impacts); population declines (level 2 impacts); local or global extinctions (level 3 impacts). Magnitudes for negative impacts on good quality of life are classified according to the SEICAT approach^15^ in four levels, namely: no impact found (level 0); human activities are more difficult (level 1 impacts); some people stop certain activities (level 2 impacts); and an activity is locally abandoned (level 3 impacts). Positive impacts on nature (native species) or good quality of life are not assigned a magnitude in the dataset. There is no standardised methodology for classifying impact magnitudes on nature’s contributions to people since these can be measured with various indicators. We include nature’s contributions to people’s impact magnitudes as free text descriptions to allow dataset users to evaluate the information for their purposes. Positive and negative impacts of the same invasive alien species at the same location and time on different entities (e.g., native species, groups of people) are reported separately in GIDIAS.

### Data recording

For each impact record, we extracted data from sources for the following variables (Supplementary Materials 2):

- Information about the invasive alien species, including its scientific name, its taxonomic classification according to the Global Biodiversity Information Facility (GBIF, https://www.gbif.org), its functional group, and its native range (if known);
- Information about the assessor and the data source, including the bibliographic reference and/or the digital object identifier (DOI); text excerpt describing the impact; year of reference; year of impact (if known); language of the source; type of source; and methodology used to infer the impact;
- Information about the impact location, including the IPBES region in which the impact was observed; the country (or sub-region for large countries or if the location was spatially disconnected from the main country area); whether the impact was on an island (yes,no) and/or in a protected area (yes,no); realm; the habitat type (classified as IPBES unit of analysis); and the spatial scale at which the impact was described;
- Information about the impact on nature, including the affected native species; affected ecosystem property; investigated level of organisation; EICAT mechanism; impact direction; impact magnitude; and whether or not the impact led to a global extinction of a native species;
- Information about the impact on nature’s contributions to people, including details of the category of nature’s contributions to people impacted (see Table 1); impact direction; and impact magnitude;
- Information about impacts on good quality of life, including the affected constituents of well-being; impact direction; impact magnitude; and whether the impact is relevant for indigenous people and local communities (IPLC).

Each impact record received a consecutive number (row number) and a unique identification indicating the original dataset (usually a region/taxon combination) it originated from (uniqueID).

### Data standardisation and merging to produce final dataset

Production of the final impacts dataset involved five stages of data standardisation and validation: Step 1: preparing individual datasets as machine-readable files and merging 37 datasets in R Statistical Software (https://www.R-project.org, version 4.3.1); Step 2: standardising variables; Step 3: removing duplicates (i.e. the same impacts recorded independently by different assessors); Step 4; joining with approved GBIF taxonomy; Step 5: validation by different members of the core author team (i.e. SB, ERC, MC, PC). No novel code was developed during the data gathering, merging, or validation stages. Cleaning and merging tasks were carried out with standard functions available, including R packages *tidyverse*^30^, *janitor* (https://cran.r-project.org/package=janitor), and *readr* (https://cran.r-project.org/package=readr). Steps 1–3 are described here, for Steps 4-5 see “Technical Validation”.

Steps 1&2: All 37 input datasets were converted from Excel to CSV files, which involved standard data curation tasks (e.g., harmonising variable names, removing commas). All input datasets were then merged into one dataset, and each variable was assigned standard factor levels for those variables with predefined levels (see Supplementary Materials 2; e.g. affected nature’s contribution to people, type of source); and free text for all other variables (see Supplementary Materials 2; e.g. text excerpt, methodology details). The variables of ‘unit of analysis’ (referring to habitat type), ‘affected nature’s contributions to people’, and ‘affected constituent of well-being’ were retained as entered and additionally converted to binary factors of each level in wide dataset format (i.e., TRUE, FALSE) to facilitate analysis, particularly as some impact records could occur across multiple levels of these variables (i.e., affects multiple habitat types).

Step 3: Given that some literature searches were global and others regional and were carried out independently by different assessors, we checked the dataset for duplicate records of the same impacts from the same source. Suspected duplicates were identified by comparing the reference, year, and other core variables across different assessors and then manually checked by the core author team. Confirmed duplicates were then removed from the dataset.

## Data Records

The GIDIAS impacts dataset, metadata and a document containing the Supplementary Materials are stored on Figshare^31^.

The dataset is provided in a machine-readable CSV file, with special language characters retained where used (UTF-8 format). The dataset is also provided in Excel format. Metadata is provided in Excel format, including descriptors for each variable.

## Technical Validation

Standardised, reliable, and unbiased collection of impact data was ensured by splitting the world into continental regions, including some further sub-regions, and further into the major taxonomic groups (plants, vertebrates, invertebrates, microorganisms), so that a breadth of region/taxon combinations were covered. More than 100 assessors provided impact data according to their specific regional and taxonomic expertise. To mitigate individual subjectivity, all datasets were cross-checked for adhering to the standardised notations agreed upon in the data collection template (see metadata) by at least one member of the core author team (SB, ERC, MC, PC) and who was not involved in the gathering of the specific dataset and corrected if necessary. The quality of impact classification also benefited from using global standards (IUCN EICAT, SEICAT) that are also recommended by the Convention on Biological Diversity (CBD) at their latest Conference of Parties meeting (https://www.cbd.int/documents/CBD/COP/16/L.4). The data search process followed international guidelines by the IUCN, which are based on guidelines from the Collaboration for Environmental Evidence (https://www.environmentalevidence.org/information-for-authors). Searches within several major bibliographic resources and in 16 languages ensured that the collected sample represented the current level of knowledge to the greatest extent possible.

### Standardising invasive alien species taxonomy

Scientific names of invasive alien species were recorded as assessors found them reported in the resources (IAS.Species.Name) and thus contained synonyms and outdated names. We cross-checked all names with the Global Biodiversity Information Facility (GBIF) backbone taxonomy using the *rgbif* package in R (version 3.8.0, https://cran.r-project.org/package=rgbif). We selected accepted names and exact matches where possible, but if not successful, we checked accepted names among synonyms with exact matches and checked accepted names using fuzzy matching with high confidence (>97) to account for minor spelling errors. The remaining unresolved entries were spelling errors where we added the correct information manually (339 of 3354 taxa).

Four different members of the author core team (SB, ERC, MC, PC) and an external colleague (Dr. H. Seebens, University of Giessen, Germany) checked the dataset separately to identify duplicates, check binary variables compared to original columns (which were retained for comparison), and check the standardised levels of variables. The responsible assessor from each dataset was asked to check their dataset for duplicates that were identified, which involved going back to original sources and checking data had been entered in a standardised way and checking that the final merged dataset contained the same information as the local dataset provided by the assessor.

### Coverage

The GIDIAS has 22865 impact reports, including 13221 impacts on nature, 7232 impacts on nature’s contributions to people, and 3302 impacts on good quality of life (Table 3). Note that the same invasive alien species can affect multiple impact types (Nature, Nature’s Contributions to People, Good Quality of Life) at the same location and point in time. The GIDIAS includes impact reports from 172 of all 195 countries (total country coverage 88%). The highest coverage is for African and North American countries (96% and 91% coverage, respectively) while the lowest coverage is for Oceanian countries (64%) (Table 2).

**Table 2:** Number and percentage of countries with impact reports and numbers of impact records on nature, nature’s contributions to people, and good quality of life per continent in GIDIAS.

Continent/region	Countries with impact records	Percentage of countries with impact records	Impacts on nature	Impacts on nature’s contributions to people	Impacts on good quality of life
Africa	52	96%	1157	600	431
Asia	42	88%	1569	914	1062
Europe	38	86%	4323	3222	812
North America	21	91%	3354	1613	660
Oceania	9	64%	1566	362	220
South America	10	83%	1252	521	117
**Total**	**172**	**88%**	**13221**	**7232**	**3302**

Note that the same impact can affect more than one impact type.

Most impact records are from Europe and Central Asia (7865), the Americas (7806), and the Asia-Pacific region (5338), while fewer records are documented from Africa (1719), and only 6 impact records from the Antarctic. 131 impacts were not assigned to a region because they either concern alien species without any impact reports or had impacts in multiple regions. In all regions, most impacts are recorded from the terrestrial realm (Fig. 1), but aquatic realms are represented with several hundred impact records in each region. All major taxonomic groups are well represented in all regions, except the microorganisms and the Antarctic region which are understudied (Table 3).

**Table 3:** Number of distinct invasive alien species with recorded impacts by IPBES region and taxon in GIDIAS.

IPBES Region	Plants	Vertebrates	Invertebrates	Microorganisms
Africa	147	62	76	24
Americas	572	174	390	54
Asia Pacific	366	232	260	64
Europe & Central Asia	220	114	1264	22
Antarctica	1	2	0	0
**Total**	**1020**	**447**	**1752**	**140**

## Usage Notes

When interpreting invasive alien species’ impacts, care should be taken to examine them in a comprehensive manner, addressing nature, nature’s contributions to people, good quality of life, and their directionality. Reporting all types of impacts separately, both positive and negative, allows a more nuanced and detailed foundation from which to then synthesise trends^32^, rather than tallying or calculating “net impacts” as baseline data, which may mask impacts on certain societal or environmental components. For example, economic benefits are often gained by a small sector of society, while costs, often long-term ones, are borne by the wider public^33–35^.

When using the dataset to search for recorded impacts, first ensure the dataset is filtered for records with at least one impact (i.e. filled impact fields for either nature, nature’s contribution to people or good quality of life), as there are some alien species that were searched during the checklist strategy where no impact was found, and still remain part of the dataset (615 out of 22865 records), or some alien species for which a neutral impact was recorded, or no directionality was recorded (e.g. ecosystem properties).

## Supplementary information

Supplementary Materials for Global Impacts Dataset of Invasive Alien Species (GIDIAS)

## Data Availability

No custom code was used.
