# Correction: Global Impacts Dataset of Invasive Alien Species (GIDIAS)

**DOI:** 10.1038/s41597-025-05750-x

**Published:** 2025-08-08

**Authors:** Sven Bacher, Ellen Ryan-Colton, Mario Coiro, Phillip Cassey, Bella S. Galil, Martin A. Nuñez, Michael Ansong, Katharina Dehnen-Schmutz, Georgi Fayvush, Romina D. Fernandez, Ankila J. Hiremath, Makihiko Ikegami, Angeliki F. Martinou, Shana M. McDermott, Cristina Preda, Montserrat Vilà, Olaf L. F. Weyl, Neelavar Ananthram Aravind, Ioanna Angelidou, Katerina Athanasiou, Vidyadhar Atkore, Jacob N. Barney, Tim M. Blackburn, Eckehard G. Brockerhoff, Clinton Carbutt, Luca Carisio, Pilar Castro-Díez, Vanessa Céspedes, Aikaterini Christopoulou, Diego F. Cisneros-Heredia, Meghan Cooling, Maarten de Groot, Jakovos Demetriou, James W. E. Dickey, Virginia G. Duboscq-Carra, Regan Early, Thomas G. Evans, Paola T. Flores-Males, Belinda Gallardo, Monica Gruber, Cang Hui, Jonathan M. Jeschke, Natalia Z. Joelson, Mohd Asgar Khan, Sabrina Kumschick, Lori Lach, Katharina Lapin, Simone Lioy, Chunlong Liu, Zoe J. MacMullen, Manuela A. Mazzitelli, John Measey, Agata A. Mrugała-Koese, Camille L. Musseau, Helen F. Nahrung, Alessia Pepori, Luis R. Pertierra, Elizabeth F. Pienaar, Petr Pyšek, Gonzalo Rivas Torres, Henry A. Rojas Martinez, Julissa Rojas-Sandoval, Ned L. Ryan-Schofield, Rocío M. Sánchez, Alberto Santini, Davide Santoro, Riccardo Scalera, Lisanna Schmidt, Tinyiko Cavin Shivambu, Sima Sohrabi, Elena Tricarico, Alejandro Trillo, Pieter van’t Hof, Lara Volery, Tsungai A. Zengeya

**Affiliations:** 1 Department of Biology, University of Fribourg, Fribourg, Switzerland; 2 Research Institute for the Environment and Livelihoods, Charles Darwin University, Alice Springs, Australia; 3 Senckenberg Research Institute and Natural History Museum, Frankfurt am Main, Germany; 4 School of Biological Sciences, University of Adelaide, Adelaide, SA 5000 Australia; 5 Steinhardt Museum of Natural History and Israel National Center for Biodiversity Studies, Tel Aviv University, Tel Aviv, Israel; 6 Department of Biology and Biochemistry, University of Houston, Houston, TX USA; 7 Department of Silviculture and Forest Management, Kwame Nkrumah University of Science and Technology, Kumasi, Ghana; 8 Centre for Agroecology, Water and Resilience, Coventry University, Ryton Gardens, Coventry, CV8 3LG UK; 9 Institute of Botany after A. Takhtajyan NAS RA, Yerevan, Armenia; 10 Instituto de Ecología Regional, Universidad Nacional de Tucumán-CONICET, Yerba Buena, Tucumán, Argentina; 11 Ashoka Trust for Research in Ecology and the Environment (ATREE), Srirampura, Jakkur Post, Bangalore, 560064 India; 12 Lake Biwa Branch Office, National Institute for Environmental Studies, Otsu, Shiga Japan; 13 Climate and Atmosphere Research Center (CARE-C), The Cyprus Institute, Nicosia, Cyprus; 14 Department of Economics, University of Missouri, Columbia, MO USA; 15 Department of Natural Sciences, Ovidius University of Constanta, Constanta, Romania; 16 Departamento de Biología Vegetal y Ecología, Universidad de Sevilla, 41092 Sevilla, Spain; 17 Estación Biológica de Doñana, EBD-CSIC, Sevilla, Spain; 18 South African Institute for Aquatic Biodiversity, Grahamstown, South Africa; 19 Joint Services Health Unit, British Forces, Cyprus; 20 Salim Ali Centre for Ornithology and Natural History (SACON), Coimbatore, India; 21 Virginia Tech /, Blacksburg, VA USA; 22 Centre for Biodiversity and Environment Research, Department of Genetics, Evolution and Environment, University College London, London, UK; 23 Institute of Zoology, Zoological Society of London, London, UK; 24 Swiss Federal Research Institute WSL, Birmensdorf, Switzerland; 25 School of Life Sciences, University of KwaZulu-Natal, Scottsville, 3209 South Africa; 26 Istituto Zooprofilattico Sperimentale del Piemonte Liguria e Valle d’Aosta, Torino, Italy; 27 Department of Life Sciences, University of Alcalá, Alcalá, Spain; 28 Ecology Aquatic and Microscopy Laboratory, Estación Biológica de Doñana, EBD-CSIC, Sevilla, Spain; 29 Department of Ecology and Systematics, Faculty of Biology, National and Kapodistrian University of Athens, Athens, Greece; 30 Colegio de Ciencias Biológicas y Ambientales, Universidad San Francisco de Quito (USFQ), Quito, Ecuador; 31 Pacific Biosecurity / Victoria University of Wellington, Wellington, New Zealand; 32 Slovenian Forestry Institute, Ljubljana, Slovenia; 33 Leibniz-Institute of Freshwater Ecology and Inland Fisheries (IGB), Berlin, Germany; 34 Freie Universität Berlin, Berlin, Germany; 35 GEOMAR Helmholtz Centre for Ocean Research, Kiel, Germany; 36 CONICET Patagonia Norte, Universidad Nacional de la Plata, La Plata, Argentina; 37 Centre for Ecology and Conservation, University of Exeter, Penryn Campus, Cornwall, UK; 38 Instituto Pirenaico de Ecologia (IPE), Spanish National Research Council (CSIC), Zaragoza, Spain; 39 Te Herenga Waka—Victoria University of Wellington, Wellington, New Zealand; 40 Centre for Invasion Biology, Department of Mathematical Sciences, Stellenbosch University, Stellenbosch, South Africa; 41 Georg-August University of Goettingen, Goettingen, Germany; 42 Department of Botany, University of Kashmir, Srinagar, India; 43 Centre for Invasion Biology, Department of Botany and Zoology, Stellenbosch University, Stellenbosch, South Africa; 44 South African National Biodiversity Institute, Kirstenbosch Research Centre, Kirstenbosch, South Africa; 45 Centre for Tropical Biosecurity, James Cook University, Cairns, Australia; 46 Austrian Research Centre for Forests, Vienna, Austria; 47 Department of Agricultural, Forest and Food Sciences, University of Turin, Grugliasco, Italy; 48 Key Laboratory of Mariculture, Ministry of Education, College of Fisheries, Ocean University of China, Qingdao, China; 49 ICPA, Universidad de Tierra del Fuego, Ushuaia, Argentina; 50 Institute of Biodiversity, Yunnan University, Kunming, China; 51 Forest Research Institute / University of the Sunshine Coast, Brisbane, Australia; 52 Institute for Sustainable Plant Protection - C.N.R, Sesto Fiorentino, Italy; 53 National Museum of Natural Sciences (CSIC-MNCN), Madrid, Spain; 54 Warnell School of Forestry and Natural Resources, University of Georgia, 180 E. Green Street, Athens, Georgia USA; 55 Institute of Botany, Czech Academy of Sciences, Průhonice, Czech Republic; 56 Estación de Biodiversidad Tiputini, Universidad San Francisco de Quito, Quito, Ecuador; 57 Institute of the Environment & Department of Geography, Sustainability, Community, and Urban Studies, University of Connecticut, Storrs, Connecticut USA; 58 Instituto de Diversidad y Ecología Animal, Universidad Nacional de Córdoba-CONICET, Córdoba, Argentina; 59 National Research Council, Institute for Sustainable Plant Protection, Sesto fiorentino, Italy; 60 Ministry for Primary Industries, Biosecurity New Zealand /, Wellington, New Zealand; 61 IUCN/SSC Invasive Species Specialist Group, Rome, Italy; 62 Estonian Academy of Arts, Tallinn, Estonia; 63 Department of Environmental Sciences, University of South Africa, Roodepoort, South Africa; 64 Ferdowsi University of Mashhad, Gorgan, Iran; 65 Department of Biology, University of Florence, Sesto Fiorentino, FI Italy; 66 Centre for Invasion Biology, Department of Zoology and Entomology, University of Pretoria, Pretoria, South Africa

**Keywords:** Invasive species, Interdisciplinary studies

Correction to: *Scientific Data* 10.1038/s41597-025-05184-5, published online 21 May 2025

In this article the author’s name Thomas G. Evans was incorrectly written as Thomas E. Evans. The original article has been corrected.

