# Supplementary material for: Global Impacts Dataset of Invasive Alien Species (GIDIAS)
Source: Sci Data. 2025 May 21;12:832. doi: 10.1038/s41597-025-05184-5 (PMC12095621; doi:10.1038/s41597-025-05184-5)
Supplement: Supplementary file 1 — Supplementary Materials for Global Impacts Dataset of Invasive Alien Species (GIDIAS) [file 41597_2025_5184_MOESM1_ESM.pdf]

Supplementary Materials for Global Impacts Dataset of Invasive Alien Species (GIDIAS)

Table of contents

Supplementary Materials 1: Environmental and Socio-Economic Impact Classification for Alien Taxa (EICAT, SEICAT) 3

Supplementary Materials 2: Description of the variables included in the Global Impacts Dataset of Invasive Alien Species GIDIAS 5

Supplementary Materials 3: Search strategies and datasets included in the Global Impact Dataset of Invasive Alien Species (GIDIAS) 7

- Forest pathogens/Europe and Central Asia 7
- Ungulates/global 7
- Chytrid fungus, Rosy wolf snail/global 7
- All taxa/China 8
- Birds/global 8
- Amphibians/global 8
- Rodents/global 9
- Reptiles/Europe and Central Asia 9
- Fish (freshwater)/Europe and Central Asia 9
- Plant and microbes/South America 10
- Plants/Galapagos 10
- Marine/global 11
- Plants/Europe and Central Asia 12
- Plants (Aquatic plants)/Europe and Central Asia 12
- Plants (impacts on hybridization)/Europe and Central Asia 13
- Plants (impacts on NCP and GQL)/Europe and Central Asia 13
- All taxa/Russian Federation (Siberia, Far East, Caucasus), Central Asia, South Caucasus 14
- Freshwater invertebrates/Europe and Central Asia 15

Terrestrial invertebrates/Europe and Central Asia 15
Plants /Africa 16
Aquatic plants/Africa 16
Vertebrates/Africa 16
Vertebrates/Africa 17
Fish and crayfish/Africa 17
Animals/North America, Mesoamerica, Caribbean 18
All taxa/East Asia, South-East Asia 18
All taxa/East Asia 19
All taxa / West Asia 19
Molluscs/South Asia 19
Plants/Iran 20
Fish/South Asia 20
Multiple taxa/South Asia 21
Plants, microbes, invertebrates/Oceania 22
Ants/Global 23
Plants/Caribbean 24
Plants/North America 24
Vertebrates/Galapagos 25
Invertebrates/Galapagos 25
Vertebrates/Asia Pacific 26
Literature cited 33

Supplementary Materials 1: Environmental and Socio-Economic Impact Classification for Alien Taxa (EICAT, SEICAT)

The International Union for Conservation of Nature (IUCN) Environmental Impact Classification for Alien Taxa (EICAT) framework was developed to categorize and assess negative impacts caused by alien taxa to native taxa (IUCN, 2020). The basic idea behind the framework is to assess how much a native species is affected by an invasive alien species. Other types of environmental impacts such as changes caused by alien taxa to abiotic ecosystem properties (e.g., soil or water chemistry) are considered under the framework only if such changes lead to a decrease in attributes of native biodiversity.

The EICAT classifies impacts in a 5-step semi-quantitative scale based on the level of biological organisation affected (individuals → populations → communities), and the magnitude and reversibility of these impacts (Blackburn et al., 2014). The five steps reflect an increase in the order of magnitude of the particular impact so that a new level of biological organisation is involved. **Minimal Concern** – negligible impacts, and no reduction in performance of a native taxon's individuals; **Minor** – performance of individuals reduced, but no decrease in population size; **Moderate** – native taxon population decline; **Major** – native taxon local extinction (i.e., change in community structure), which is naturally reversible; and **Massive** – naturally irreversible local or global extinction of a native taxon (IUCN, 2020; Volery et al., 2020). Impacts of invasive alien species can be caused through 10 mechanisms. The EICAT is conceptually and structurally related to the IUCN Red List of Threatened Species, with the Red List categorizing a focal native species based on its risk of extinction, and the EICAT categorizing a focal alien taxon based on the degree to which it has negatively impacted native taxa (Van der Colff et al., 2020).

The Socio-Economic Impact Classification for Alien Taxa (SEICAT) assesses negative impacts of invasive alien species on good quality of life (Bacher et al., 2018). It follows an approach similar to the EICAT. In particular, it classifies changes in human activities caused by invasive alien species into one of 5 magnitudes. These are: **Minimal Concern** – negligible impacts, and no reduction in individual peoples' activities; **Minor** – normal activities are more difficult, but no decrease in activity size, i.e., all people still carry out the activity; **Moderate** – decline in activity size, i.e., fewer people participate in an activity; **Major** – local disappearance of an activity from all or part of the area invaded by the invasive alien species, which is naturally reversible; and **Massive** – local irreversible disappearance of an activity from all or part of the area invaded by the invasive alien species (Bacher et al., 2018). Changes in human activities can be caused through impacts on five constituents of good quality of life. The framework is based on the capability approach of welfare economics (Robeyns, 2005; Sen, 1999) and thus avoids ambiguities in interpreting impacts based on monetary approaches (Hoagland & Jin, 2006).

The EICAT and the SEICAT have been used to compare impact magnitudes of alien taxa at various spatial scales, across geographic regions and taxonomic groups (e.g., Evans et al., 2016, 2020; Canavan et al., 2019; Galanidi et al., 2018; Kesner & Kumschick, 2018; Volery et al., 2021), and to facilitate evidence-based prioritization and other management decisions (Rockwell-Postel et al., 2020). Widespread application of both schemes is expected to reduce the data biases and data gaps on the impacts of invasive alien species on nature and good quality of life. Recently, the EICAT framework was expanded to include a classification for positive impacts of invasive alien species for nature (Vimercati et al., 2022) but this was not available at the time when data for this dataset were gathered. EICAT+ might allow comparison of positive and negative environmental impacts in a common framework for a better understanding of the consequences of biological invasions and

to better inform conservation decisions. For a comprehensive understanding and efficient management, the reporting of both negative and positive impacts is critical (Vimercati et al., 2020).

Supplementary Materials 2: Description of the variables included in the Global Impacts Dataset of Invasive Alien Species GIDIAS

Variable category	No. of variables	Variable names	Example of variables
Identifiers	2	rowID; UniqueID	Unique identifier of impact records, indicating the input dataset (22865 entries)
Invasive alien species	12	IAS.Species.Name; Verified.Name.GBIF.Taxon;GBIF.scientificName.with.author; Taxonomic levels (Genus to Kingdom); IAS.Taxon; IAS.Functional.Group; native.range.of.IAS	Name of invasive alien species as assigned by the assessor (3779 entries); GBIF Taxonomy; Larger taxonomic group of invasive alien species (4 levels): plant, invertebrate, vertebrate, microbe; Native range of invasive alien species (if available) according to assessor (free text)
Source	10	Reference; Text.excerpt; DOI; Assessor; Year; Year.of.impact; Type.of.source; Peer.reviewed; Language; Methodology.details	Source (citation) describing the impact (if given) (6783 entries); Verbatim excerpt of the text describing the impact; Year of impact, if different from the publication year (1600-2019); Type of source document describing the impact (15 levels); Details about the method to assess impact (free text)
Location	9	Region; Country.Location; Island; Island.k; Protected.area; Protected.area.k; Realm; Units.of.Analysis.clean; Spatial.scale	IPBES region in which the impact was described (5 levels); Name of island and/or protected area for which the impact was described (free text) and binary variable for Island and/or Protected area (0,1); Realm in which the impact was observed (3 levels): Terrestrial, Marine. Freshwater; Habitat type(s) in which the impact was described (15 levels), using the IPBES Units of Analysis classification: https://www.ipbes.net/glossary/units-analysis.
Impacts on nature	9	Affected.native.species.Details; Affected.native.species.Taxon; Affected.ecosystem.property; investigated.level.of.organization; mechanism.Nature.clean; direction.Nature; direct.or.indirect.Nature; global.extinction; magnitude.Nature	Larger taxonomic group of affected native species (5 levels): Plant, Invertebrate, Vertebrate, Microbe, Fungi; IUCN EICAT mechanisms (12 levels, as listed in https://doi.org/10.2305/IUCN.CH.2020.05.en), with positive mechanisms partially classified; Impact direction (3 levels): Positive if native species profit; Neutral (partially classified); Negative if native species suffer; Whether the impact led to a global extinction of a native species; EICAT magnitudes (4 levels): level to which a native species is suffering from the invasive alien species

Impacts on nature's contributions to people	4	affected.NCP.clean; details.NCP; direction.NCP; magnitude.NCP	IPBES categories of Nature's Contributions to People (18 levels, as described in https://www.science.org/doi/suppl/10.1126/science.aap8826/suppl_file/aap8826-diaz-sm.pdf); Indicates whether the invasive alien species changed nature's contributions to people (s) to the benefit or detriment of people (3 levels): Positive, Neutral (partially classified), Negative; Details about the described impact magnitude on nature's contributions to people (free text)
Impacts on Good Quality of Life	6	affected.CWB.clean; details.CWB; direction.CWB; magnitude.CWB; magnitude.CWB.details; IPLC	Affected constituents of well-being according to the SEICAT approach (5 levels, as described in https://doi.org/10.1111/2041-210X.12844); Details about the described impact on CWB (free text); Impact magnitude on peoples' activities being according to the SEICAT approach (5 levels including 4 negative and 1 positive), adapted from https://doi.org/10.1111/2041-210X.12844); Whether the impact involves Indigenous People and Local Communities and/or is sourced from Indigenous and Local Knowledge
Binary variables	38	UoA.Dry; UoA.Boreal NCP:Habitat; NCP.Pollination; CWB.Safety; CWB.Assets and so on	Binary variables for each level of Unit of Analysis (habitat type), nature's contribution to people and constituent of well-being affected (0,1)

Supplementary Materials 3: Search strategies and datasets included in the Global Impact Dataset of Invasive Alien Species (GIDIAS)

Forest pathogens/Europe and Central Asia

Assessor(s) – Alberto Santini, Alessia Pepori

Search language(s) – English

Search terms – TITLE-ABS-KEY ("species name" AND impact AND (portugal OR spain OR france OR "Great Britain" OR ireland OR italy OR germany OR switzerland OR poland OR "Czech Republic" OR slovakia OR slovenia OR croatia OR serbia OR ukraine OR russia OR georgia OR turkey OR azerbaijan OR armenia OR belgium OR Iran OR “The Netherlands” OR Luxembourg OR Belarus))

Search engine – WoS

Date – March 2020

Overview of the search results – 190 records of impacts were obtained

Unique Identifier Prefix – EU_Pathogens

Ungulates/global

Assessor(s) – Lara Volery, Sven Bacher, Mario Coiro

Search language(s) – English

Search terms – (‘introduced’ OR ‘invasive’ OR ‘alien’ OR ‘non-native’ OR ‘non-indigenous’ OR ‘feral’ OR ‘exotic’ AND ‘[common name]´ OR ‘[latin name]’)

Search engine – GoogleScholar, WoS, UCL Explore (https://www.ucl.ac.uk/library/electronic-resources/about-explore), the CABI’s Invasive Species Compendium (ISC; https://www.cabi.org/ISC) and Google (https://www.google.ch)

Date – May 2020

Results were published in Volery et al. 2021

Overview of the search results – 334 records of impacts were obtained

Unique Identifier Prefix –IPBES_EUROPE_VERT

Chytrid fungus, Rosy wolf snail/global

Assessor(s) – Sven Bacher

Search language(s) – English

Search terms – (‘introduced’ OR ‘invasive’ OR ‘alien’ OR ‘non-native’ OR ‘non-indigenous’ OR ‘exotic’ AND ‘[chitrid]´ OR ‘[Batrachochytrium dendrobatidis]’)

(‘introduced’ OR ‘invasive’ OR ‘alien’ OR ‘non-native’ OR ‘non-indigenous’ OR ‘exotic’ AND ‘[rosy wolf snail]´ OR ‘[Euglandina rosea]’)

Search engine – GoogleScholar, WoS, the CABI’s Invasive Species Compendium (ISC; https://www.cabi.org/ISC) and Google (https://www.google.ch)

Date – May 2021

Overview of the search results – 31 records of impacts were obtained

Unique Identifier Prefix – Chitrids

All taxa/China

Assessor(s) – Cang Hui, Xiaozhuo Han

Search language(s) – Chinese

Search terms – 外来种 危害 , 入侵植物 危害, 外来种 多样性, 外来种 化感作用, 入侵种 化感 (translation: Alien species, Invasive plant impact, Exotic species diversity, Allelopathy, Invasive species allelopathy)

Search engine –CNKI (www.cnki.net)

Date – April 2021

Overview of the search results – 59 records of impacts were obtained

Unique Identifier Prefix – Asia_bacteria, Asia_invertebrate, Asia_plant

Birds/global

Assessor(s) – Tom Evans, Mario Coiro, Sven Bacher

We included all original references from the global reviews on environmental and socio-economic impacts of birds from Evans et al. 2016, 2020.

Overview of the search results – 399 records of impacts were obtained

Unique Identifier Prefix – IPBES_EUROPE_VERT

Amphibians/global

Assessor(s) – John Measey, Mario Coiro, Sven Bacher

Search language(s) – English, French, Spanish

We included all original references from the global reviews on environmental and socio-economic impacts of amphibians from Measey et al. 2016

Overview of the search results – 356 records of impacts were obtained

Unique Identifier Prefix – IPBES_EUROPE_VERT

Rodents/global

Assessor(s) – Lisanna Schmidt, Johann Cheseaux, Maddie Harris, Mario Coiro, Sven Bacher

Search language(s) – English, French, Spanish, German

Search terms – (‘introduced’ OR ‘invasive’ OR ‘alien’ OR ‘non-native’ OR ‘non-indigenous’ OR ‘feral’ OR ‘exotic’ AND ‘[common name]´ OR ‘[latin name]’)

Search engine – GoogleScholar, WoS, and Google (https://www.google.ch)

Date – May 2020-May 2021

Overview of the search results – 289 records of impacts were obtained

Unique Identifier Prefix – IPBES_EUROPE_VERT

Reptiles/Europe and Central Asia

Assessor(s) – Riccardo Scalera

Search language(s) – English

Search terms – The assessor used the species listed in the database by Capinha et al. 2017 as a starting point, then checked private literature collected over the years, and eventually did a simple Google search species by species + relevant country/area

Search engine – GoogleScholar and Google

Date – December 2020- January 2021

Overview of the search results – 14 records of impacts were obtained

Unique Identifier Prefix – IPBES_EUROPE_VERT

Fish (freshwater)/Europe and Central Asia

Assessor(s) – Baptiste Michel, Sven Bacher, Mario Coiro

Search language(s) – English

Search terms – species list from EASIN - European Alien Species Information Network (https://easin.jrc.ec.europa.eu/easin; accessed 2019), search terms: (‘introduced’ OR ‘invasive’ OR ‘alien’ OR ‘non-native’ OR ‘non-indigenous’ OR ‘exotic’ AND ‘[common name]´ OR ‘[latin name]’)

Search engine – GoogleScholar and Google

Date – December 2019 - January 2021

Overview of the search results – 289 records of impacts were obtained

Unique Identifier Prefix – IPBES_EUROPE_VERT

Plant and microbes/South America

Assessor(s) – Romina Fernandez

Search language(s) – English, Spanish and Portuguese

Search terms –

Plants: (Invasive plant OR exotic plant OR alien plant OR no-native plant OR invasive plants) AND (impact OR effect OR increase OR decrease) AND (diversity OR abundance OR cover OR ecosystem process OR well-being OR economic OR production OR biosecurity) AND (Argentina OR Chile OR Uruguay OR Brazil OR Bolivia OR Peru OR Colombia OR Venezuela OR Paraguay OR Ecuador OR Guyana OR Surinam).

Microbes: (Invasive bacteria OR invasive fungi OR invasive microbes) AND (impact OR effect OR increase OR decrease) AND (diversity OR abundance OR cover OR ecosystem process OR well-being OR economic OR production OR biosecurity) AND (Argentina OR Chile OR Uruguay OR Brazil OR Bolivia OR Peru OR Colombia OR Venezuela OR Paraguay OR Ecuador OR Guyana OR Surinam).

The assessor also searched the references contained in the sources obtained from the literature search.

Search engine – Google Scholar, Scopus, WoS

Date – September 2019 - April 2021

Overview of the search results – 820 records of impacts were obtained

Unique Identifier Prefix – SA_plant, South_america_plantmic

Plants/Galapagos

Assessor(s) – Paola Flores-Males, Gonzalo Rivas-Torres, Pieter van `t Hof

Search language(s) – English and Spanish

Search terms – [multiple searches, separated by commas] galapagos AND non native species, galapagos AND non native species OR alien taxa OR introduced OR introduction OR invasor, galapagos AND non native species OR alien taxa OR introduced OR introduction OR invasor AND plants OR flora AND impact, galapagos AND non native species OR alien taxa OR introduced OR introduction OR invasor AND plants OR flora AND impact AND Fabaceae, galapagos AND non native species AND invasor* OR impact* , galapagos AND non native species AND invas* OR impact* AND flora OR plant* , "Bauhinia variegate" AND galapagos AND impact, "Abrus precatorius" AND galapagos AND impact, "Arachis hypogaea" AND galapagos AND impact, "Arachis pintoi" AND galapagos AND impact, "Cajanus cajan" AND galapagos AND impact, "Canavalia dictyota" AND galapagos AND impact, "Canavalia ensiformis" AND galapagos AND impact, "Canavalia rosea" AND galapagos AND impact, "Centrolobium paraense" AND galapagos AND impact, "Clitoria ternatea" AND galapagos AND impact, "Crotalaria retusa" AND galapagos AND impact, "Desmodium glabrum" AND galapagos AND impact, "Desmodium incanum" AND galapagos AND impact, "Desmodium intortum" AND galapagos AND impact, "Desmodium limense" AND galapagos AND impact, "Dioclea reflexa" AND galapagos AND impact, "Dioclea virgata" AND galapagos AND impact, "Erythrina corallodendron" AND galapagos AND impact, "Erythrina edulis" AND galapagos AND impact, "Erythrina fusca" AND galapagos AND impact, "Erythrina poeppigiana" AND galapagos AND impact, "Erythrina smithiana" AND galapagos AND impact, "Galactia tenuiflora" AND galapagos AND impact, "Geoffroea spinosa" AND galapagos AND impact, "Gliricidia sepium" AND galapagos

AND impact, “Glycine max” AND galapagos AND impact, “Indigofera suffruticosa” AND galapagos AND impact, “Lablab purpureus” AND galapagos AND impact,"Lens culinaris" AND galapagos AND impact,"Macroptilium lathyroides" AND galapagos AND impact,"Medicago sativa" AND galapagos AND impact,"Mucuna rostrata" AND galapagos AND impact,"Phaseolus coccineus" AND galapagos AND impact,"Phaseolus lunatus" AND galapagos AND impact,"Phaseolus vulgaris" AND galapagos AND impact,"Pisum sativum" AND galapagos AND impact,"Pueraria phaseoloides" AND galapagos AND impact,"Spartium junceum" AND galapagos AND impact,"Vicia faba" AND galapagos AND impact,"Vigna unguiculata" AND galapagos AND impact,"Zornia curvata" AND galapagos AND impact,"Zornia piurensis" AND galapagos AND impact,"Bauhinia monandra" AND galapagos AND impact,"Caesalpinia bonduc" AND galapagos AND impact,"Caesalpinia gilliesii" AND galapagos AND impact,"Caesalpinia pulcherrima" AND galapagos AND impact,"Cassia fistula" AND galapagos AND impact,"Cassia grandis" AND galapagos AND impact,"Delonix regia" AND galapagos AND impact,"Schizolobium parahyba" AND galapagos AND impact, "Senna alata" AND galapagos AND impact,"Senna bicapsularis" AND galapagos AND impact,"Senna hirsuta" AND galapagos AND impact,"Senna obtusifolia" AND galapagos AND impact,"Senna septemtrionalis" AND galapagos AND impact,"Senna siamea" AND galapagos AND impact,"Tamarindus indica" AND galapagos AND impact, "Albizia guachapele" AND galapagos AND impact,"Acacia caven" AND galapagos AND impact,"Acacia nilotica" AND galapagos AND impact,"Calliandra calothyrsus" AND galapagos AND impact,"Inga edulis" AND galapagos AND impact,"Inga insignis" AND galapagos AND impact, Inga sapindoides AND galapagos AND impact, “Inga spectabilis” AND galapagos AND impact, “Inga striata” AND galapagos AND impact, “Inga vera” AND galapagos AND impact, “Leucaena leucocephala” AND galapagos AND impact, “Leucaena trichodes” AND galapagos AND impact, “Mimosa pudica” AND galapagos AND impact, “Ochroma pyramidale” AND galapagos AND impact, “Cleome viscosa" AND galapagos AND impact, “Cucumis dipsaceus” AND galapagos AND impact, “Ricinus communis” AND galapagos AND impact, “Brachiaria mutica” AND galapagos AND impact, “Digitaria eriantha” AND galapagos AND impact, “Citrus x aurantiifolia” AND galapagos AND impact, Citrus x limon AND galapagos AND impact, “Citrus medica” AND Galapagos AND impact, “Datura stramonium” AND galapagos AND impact, “Solanum lycopersicum” AND Galapagos AND impact, “Trema micrantha” AND galapagos AND impact, “Furcraea hexapetala” and Galapagos, “Cordia alliodora” AND Galapagos, “Tradescantia fluminensis” AND Galapagos, “Bryophyllum pinnatum invasive” AND Galapagos, “Persea americana invasive” AND Galapagos, “Cedrela odorata invasive” AND Galapagos, “Psidium guajava invasive” AND Galapagos, Syzygium jambos invasive” AND Galapagos, “Passiflora edulis invasive” AND Galapagos, “Melinis minutiflora” AND Galapagos, “Panicum maximum” AND Galapagos, “Pennisetum purpureum” AND Galapagos, “Rubus niveus” AND Galapagos, “Cinchona pubescens” AND Galapagos , “Cestrum auriculatum” AND Galapagos, “Lantana camara” AND Galapagos

Search engine – Google Scholar; BioOne; COBUEC; CABI; EBSCO; ScienceDirect; ProQuest; Scopus; Scielo. Assessors also searched the references contained in the sources we obtained from the literature search.

Unique Identifier Prefix – Galapagosplants

Marine/global

Assessor(s) – Bella Galil

Search language(s) – English, Spanish, Portuguese

Search terms – "alien" “invasive” “introduced” “non-native” “non-indigenous” AND impact AND (sea (e.g. Baltic, Mediterranean), country, or “species scientific name” AND impact AND (sea (e.g.

Baltic, Mediterranean), country. References cited within resulting articles were examined, and where appropriate, were accessed and assessed.

Search engine – GoogleScholar

Date – 2019 – June 2021

Overview of the search results – about 2100 records of impacts

Unique Identifier Prefix – Marine

Plants/Europe and Central Asia

Assessor(s) – Montserrat Vilà, Pilar Castro, Alejandro Trillo

Search language(s) – All

Search terms: ISI Web of Knowledge (plant invader OR exotic plant OR alien plant OR plant invasion*) AND (impact* OR effect*) AND (community structure* OR diversity* OR ecosystem process* OR competition*)

Date – December 2019

((plant invader OR exotic plant OR alien plant OR plant invasion*) AND (impact* OR effect*) AND (community structure* OR diversity* OR ecosystem process* OR competition*)) AND (Russian Federation OR Russia OR Slovakia OR Belarus OR Moldova OR Ukraine OR Albania OR Bosnia and Herzegovina OR Croatia OR Serbia OR Montenegro OR Yugoslavia OR Armenia OR Azerbaijan OR Georgia)

((plant invader OR exotic plant OR alien plant OR non-native plant OR plant invasion*) AND (impact* OR effect*) AND (nature contribution to people OR good quality of life OR human well-being)

(plant invader OR exotic plant OR alien plant OR plant invasion*) AND (impact* OR effect*) AND (community structure* OR diversity* OR ecosystem process* OR competition*) AND (Kazakhstan OR Kyrgyzstan OR Tajikistan OR Turkmenistan OR Uzbekistan)

Search engine: ISI Web

Date – November 2020

We also searched for tree impacts on NCP by references listed in Castro-Díez, P. et al. 2019. Global effects of non-native tree species on multiple ecosystem services, Biological Reviews 94: 1477-1501. p. brv.12511. doi: 10.1111/brv.12511. Paying attention not to retrieve duplicates from previous mentioned searchers

Overview of the search results – 3283 records of impacts were obtained

Unique Identifier Prefix – Eur_plants

Plants (Aquatic plants)/Europe and Central Asia

Assessor(s) – Montserrat Vilà

Search language(s) – All

Search terms: (invas* OR alien OR non-native OR exotic) AND (lake OR river OR estuary OR wetland OR reservoir) AND (impact OR effect) AND (macrophyte OR algae OR primary producer)

Search engine – Scopus

Date – November 2020

We also screened all references listed in

Brundu, G. 2015. Plant invaders in European and Mediterranean inland waters: profiles, distribution, and threats. Hydrobiologia, 746(1), 61-79.

Hussner et al. 2017. Management and control methods of invasive alien freshwater aquatic plants: a review. Aquatic Botany, 136, 112-137.

Pieterse et al. 2009. Proceedings of the 12th European Weed Research Society Symposium August 24– 28 2009, Jyväskylä, Finland

Overview of the search results – 107 records of impacts were obtained

Unique Identifier Prefix – Eur_plants

Plants (impacts on hybridization)/Europe and Central Asia

Assessor(s) – Montserrat Vilà, Alejandro Trillo

Search language(s) – All

Search terms -

(plant invader OR invasive plant OR exotic plant OR non-native plant OR alien plant OR plant invasion*) AND (plant hybrid*) AND (impact on native OR effect on native OR harmful on native OR detriment*) (plant invader OR invasive plant OR exotic plant OR non-native plant OR alien plant OR plant invasion*) AND (hybrid* OR introgres*) AND conservation

Search engine – ISI Web

Date – February 2020

Overview of the search results – 52 records of impacts were obtained

Unique Identifier Prefix – Eur_plants

Plants (impacts on NCP and GQL)/Europe and Central Asia

Assessor(s) – Montserrat Vilà, Alejandro Trillo

Search language(s) – All

Search terms:

(invasive plant OR alien plant OR non-native plant OR exotic plant) AND (impact OR effect) AND people safety

(invasive plant OR alien plant OR non-native plant OR exotic plant) AND (impact OR effect) AND Freedom

(invasive plant OR alien plant OR non-native plant OR exotic plant) AND (impact OR effect) (invasive plant OR alien plant OR non-native plant OR exotic plant) AND (impact OR effect) AND (employment OR income OR poverty OR job OR wealth OR standard of living)

(invasive plant OR alien plant OR non-native plant OR exotic plant) AND (impact OR effect) AND heritage

We also searched for references in:

Shackelton et al 2019: The role of invasive alien species in shaping local livelihoods and human well-being: A review. J Env Man 229: 145-157.

Vaz et al. 2017. Integrating ecosystem services and disservices: insights from plant invasions. Ecosyst.

Serv. 23, 94–107.

Celesti-Grapow, L., & Ricotta, C. (2020). Plant invasion as an emerging challenge for the conservation of heritage sites: the spread of ornamental trees on ancient monuments in Rome, Italy. Biological Invasions, 1-16.

Kueffer, C., & Kull, C. A. (2017). Non-native species and the aesthetics of nature. In Impact of biological invasions on ecosystem services (pp. 311-324). Springer, Heidelberg

Howard PL 2019. Human adaptation to invasive species: A conceptual framework based on a case study: metasynthesis. Ambio 2019, 48:1401–1430. https://doi.org/10.1007/s13280-019-01297-5

Nentwig W, Mebs D & Vilà M. 2017. Impacts of Non-Native Animals and Plants to Human Health. In: Vilà M & Hulme PE (eds) Impact of biological invasions on ecosystem services. Springer, Heidelberg

Kapitza, K., Zimmermann, H., Martín-López, B., & von Wehrden, H. (2019). Research on the social perception of invasive species: a systematic literature review. NeoBiota, 43, 47.

Milanović M, Knapp S, Pyšek P, Kühn I (2020) Linking traits of invasive plants with ecosystem services

and disservices. Ecosystem Services 42: 101072. doi: 10.1016/j.ecoser.2020.101072.

Search engine – Web of Science

Date – February 2021

Overview of the search results – 152 records of impacts were obtained

Unique Identifier Prefix – Eur_plants

All taxa/Russian Federation (Siberia, Far East, Caucasus), Central Asia, South Caucasus

Assessor(s) – Georgi Fayvush

Search language(s) – Russian, English

Search terms – инвазивные виды, инвазионные виды (invasive species), Siberia, Far East, Kazakhstan, Caucasus, Uzbekistan, Tadjikistan, Kyrgyzstan, Turkmenistan, вредители леса и сельского хозяйства (forest and agricultural pests)

Search engine – GoogleScholar, WoS, e-library, Researchgate, CABInternational

Date – February-April 2021

The assessor also searched the references contained in the sources obtained from the literature search

Overview of the search results– 1700 records of invasive alien species were found, but only 36 had impact assessment, they were included in the database/

Unique Identifier Prefix – NCPGQLGEORGE

Freshwater invertebrates/Europe and Central Asia

Assessor(s) – Cristina Preda, Vanessa Céspedes, James Dickey, Belinda Gallardo, Jonathan Jeschke, Bram Koese, Chunlong Liu, Agata Mrugala, Camille Musseau, Elena Tricarico, Laura Zinnert

Search language(s) – English, Spanish, German

Search terms – 1st stage query: invasive, freshwater, Europe, impact; 2nd stage refined query: [invasive alien species name] AND (Introduce* OR inva* OR alien OR nonnative OR non-native OR exotic OR naturaliz* OR non-indigenous OR nonindigenous) AND (impact* OR effect* OR change*) AND country

Search engine – GoogleScholar, Google, WoS, Scopus, CABI's Invasive Species Compendium

Date – April 2021

we also searched the references contained in the sources we obtained from the literature search

Overview of the search results – 471 records of impacts were obtained

Unique Identifier Prefix – Eu_fresh_invert, Freshw_invert_EU

Terrestrial invertebrates/Europe and Central Asia

Assessor(s) – Angeliki F. Martinou, Ioanna Angelidou, Katerina Athanasiou, -Aikaterini Christopoulou, Jakovos Demetriou, Imogen Cavadino, Lori Lach, Douglas H Boyes, Maarten de Groot, Bjorn Beckmann, Katharina Lapin, Lara Carisio, Simon Lioy, Sara Straffon

Search language(s) – English, French, German, Russian, Greek, Italian, Spanish, Finnish

Search terms – [invasive alien species name] (invas* OR alien OR non-native OR exotic) AND (impact OR Europe OR Asia)

Search engine – Google Search, Scopus

Date – up to January 2021

Overview of the search results – 2387 records of impacts were obtained

Unique Identifier Prefix – Terr_invert_eu

Plants /Africa

Assessor(s) – Katharina Dehnen-Schmutz, Clinton Carbutt

Search language(s) – English

Search terms – ((TITLE ("introduced species" OR "invasive species" OR "invasive alien species" OR "IAS" OR "alien" OR "non-native" OR "non-indigenous" OR "invasive plant" OR "pest" OR "exotic") AND TITLE-ABS-KEY ("Africa" AND "impact"))

Date – April 2020 and January 2021

Search engine – Scopus, Google Scholar

Assessors also searched the references contained in the sources obtained from the literature search

Overview of the search results – 841 records of impacts were obtained

Unique Identifier Prefix – Africa_plants

Aquatic plants/Africa

Assessor – Julie Coetzee

Search language – English

Search terms – invasive aquatic plant impact + each African country, also the scientific and common names of invasive plants

Overview of the search results – Results were obtained for Water hyacinth – *Eichhornia/Pontederia crassipes*; Water lettuce – *Pistia stratiotes*; Parrots feather – *Myriophyllum aquaticum*; Kariba weed/giant salvinia – *Salvinia molesta*; Red water fern – *Azolla filiculoides*. No other species came up with results.

Unique Identifier Prefix – Africa_plants

Vertebrates/Africa

Assessor(s) – Michael Ansong**,** Tsungai Zengeya

Search language(s) – English

Search terms – (“vertebrate” OR “invertebrate” OR “animal” OR “mammal” OR “reptile” OR “amphibian” OR “Bird” OR “insect” OR “Rodent”) ("introduced species" OR "invasive species" OR "invasive alien species" OR "IAS" OR "alien" OR "non-native" OR "non-indigenous" OR "pest" OR "exotic") AND ("Africa" OR "Country"). Assessors also searched the references contained in the sources we obtained from the literature search.

[NOTE on “country”: Countries in Africa]

Search engine – Google Scholar, Google, Web of Science

Date – April 2020

Overview of the search results - 393 records

Unique Identifier Prefix – Africa_animal

Vertebrates/Africa

Assessor(s) – Tsungai Zengeya

Search language(s) – English

We included all original references from the global reviews on environmental and socio-economic impacts of vertebrates in Africa from the following studies:

Measey J, Davies SJ, Vimercati G, Rebelo A, Schmidt W, Turner A. 2017. Invasive amphibians in southern Africa: A review of invasion pathways. Bothalia-African Biodiversity & Conservation, 47(2): 1-12. https://doi.org/10.4102/abc.v47i2.2117

Hagen BL, Kumschick S. 2018. The relevance of using various scoring schemes revealed by an impact assessment of feral mammals. NeoBiota 38: 37-75. https://doi.org/10.3897/neobiota.38.23509

Evans T, Kumschick S, Blackburn TM. 2016. Application of the Environmental Impact Classification for Alien Taxa (EICAT) to a global assessment of alien bird impacts. Diversity and Distribution, 22: 919-931. https://doi.org/10.1111/ddi.12464

Shivambu TC, Shivambu N, Downs CT. 2020. Impact assessment of seven alien invasive bird species already introduced to South Africa. Biological Invasions 22: 1829-1847. https://doi.org/10.1007/s10530-020-02221-9;

Van Wilgen B, Zengeya TA, Richardson D. 2022. A review of the impacts of invasive alien species in South Africa. Biological Invasions 24: 27-50. https://doi.org/10.1007/s10530-021-02623-3

Marr SM, Ellender BR, Woodford DJ, Alexander ME, Wasserman RJ, Ivey P, Zengeya TA, Wely OLF. 2017. Evaluating invasion risk for freshwater fishes in South Africa. *Bothalia* 47: a2177. https://doi.org/10.4102/abc.v47i2.2177

Overview of the search results – 85 records of impacts were obtained

Unique Identifier Prefix – ZegeneyaTA_vert

Fish and crayfish/Africa

Assessor(s) – Olaf Weyl

Search language(s) – English

Search terms – Online searches were undertaken using the following search terms within a search string, in conjunction with the species scientific and common name(s): "introduced species", "invasive species", "invasive alien species", "IAS", "AIS", "NIS", "alien", "non-native", "non-indigenous", "pest", and "exotic". Lists of alien/introduced fish and crayfish species were taken from the Global Registry of Introduced and Invasive Species (GRIIS; https://www.gbif.org/dataset/search?project_id=GRIIS) for African countries.

Search engine – Google Scholar, Google, Web of Science

Date – April 2020

Overview of the search results - 120 records

Unique Identifier Prefix – AF_Fish_crayfish

Animals/North America, Mesoamerica, Caribbean

Assessor(s) – Shana McDermott and Georgia Roberts

Search language(s) – English

Search terms – General search: (invasive OR alien AND species) AND (freshwater OR marine OR ocean OR lake OR forest OR terrestrial OR aquatic) AND (vertebrate OR invertebrate OR animal) AND (North America OR United States OR Canada OR Mexico OR Caribbean OR Florida) AND (summary OR meta-analysis OR meta OR analysis) AND (effects OR impacts). Some species were also included in the search terms above: (species scientific name OR emerald ash borer OR EAB OR feral hog OR starlings OR spiny waterflea OR gypsy moth OR spotted winged drosophila OR lionfish OR northern snakehead OR monkey). Specific impacts were also included on occasion: (health OR economic OR nature OR indigenous)

Search engine – GoogleScholar

Date – October 2019 – June 2021

Assessors also searched the references contained in the sources we obtained from the literature search and included all original references from the United States/Global reviews on impacts of invasive alien species (Diagne et al. 2021; Duenas et al. 2018; Gallardo et al. 2016; Gutiérrez et al. 2014; Hanley & Roberts 2019; Howard 2019; Kapitza et al. 2019; Olson 2006; Pimentel et al. 2005; Sanderson et al. 2012; Shackleton et al. 2019).

Overview of the search results – 2,541 records of impacts were obtained

Unique Identifier Prefix – NA_vert_invert, NorthAM_carr

All taxa/East Asia, South-East Asia

Assessor(s) – Makehiko IKEGAMI

Search language(s) – English

Search terms – (("alien" or "non-native" or "non-indigenous" or "invasive" or invasion*) and ("impact" or "influence" or "effect") and ("Cambodia" or "East Asia" or "Far East" or "Indonesia" or "Japan" or "Korea" or "Laos" or "Malaysia" or "Myanmar" or "Papua New Guinea" or "Philippine" or "Singapore" or "Taiwan" or "Vietnam") NOT ("medical" or "cancer" or "drug" or "education"))

Search engine –Web of Science

Date – April 2021

Overview of the search results – 1293 results, then examined the abstract for relevance to the database

Unique Identifier Prefix – East_SEAsia_maki

All taxa/East Asia

Assessor(s) – Maki IKEGAMI

Search language(s) – Japanese

Search terms – “外来種” and “影響”with “jstage”, “alien species impact Japan” or “non-native species impact Japan” for google scholar

Search engine – https://www.jstage.jst.go.jp/ https://scholar.google.co.jp In addition, the assessor used some review papers and books (both in Japanese and English)

Date – September 2019

Overview of the search results – 2230 results were obtained from jstage, 69,300 and 35,000 results from google scholar (with “alien” and “non-native” respectively). For jstage, most relevant first 560 papers, and for google first 400 most relevant papers were examined, after 100 no relevant papers were sought. In addition, the assessor used references from the following review paper and book: Yan et al (2001) for China and JWRC(2008,2019) for Japan.

Unique Identifier Prefix – East_SEAsia_maki

All taxa / West Asia

Assessor(s) – Mohd Asgar Khan

Search language(s) – English

Search terms – (‘invasive” OR “alien” OR “exotic” OR “introduced”) AND (“insects” OR “pathogen” OR “pests” OR “parasites” OR “birds” OR “plants” OR “animals” OR “weeds” OR “reptiles” OR “amphibians”) AND (“livelihood” OR “human well-being” OR “ecosystem service” OR “diversity” OR “ecosystem function”) AND (“impacts” OR “benefits” OR “costs” OR “negative impacts” OR “positive impacts”) AND (“country name”) [NOTE on “country name”: Countries in West Asia = Bahrain, Kuwait, Oman, Qatar, Saudi Arabia, United Arab Emirates and Yemen (Arabian Peninsula); Iraq, Jordan, Lebanon, State of Palestine and Syrian Arab Republic (Mashreq)]

Search engine – Google Scholar

Date – 20 March 2021 to 22 April 2020

Criteria used to select articles to review was based on relevance of title and abstract. The assessor also used papers that were cited in literature reviews.

Overview of the search results – 40 records

Unique Identifier Prefix – WEST_ASIA

Molluscs/South Asia

Assessor(s) – N. A. Aravind Madhyastha

Search language(s) – English

Search terms – Introduced molluscs + India or South Asia Invasive molluscs + India or South Asia, “species” + India or South Asia [species = individual species name which are introduced to India or South Asia], introduced Molluscs + India/south Asia + marine

Search engine – Google Scholar

Date – April 2020

The assessor also used papers that were cited in literature reviews.

Overview of the search results - 39 records

Unique Identifier Prefix – SouthAsia_Molluscs

Plants/Iran

Assessor(s) – Sima Sohrabi

Search language(s) – English, Persian

Search terms – (1) using the species’ scientific name combined with keywords indicating its alien/invasive status and impact categories as defined in EICAT in Web of Science; (2) databases of invasive species with impacts recorded, namely CABI, GISD, USDA, and BioNET-EAFRINET.

Search engine – Web of Science (Clarivate Analytics); CABI (Invasive Species Compendium, www.cabi.org), GISD, USDA, BioNET-EAFRINET

Date – March 2021

Apart from these, bibliographic sources of information, including regional and national case studies and books (Sohrabi et al. 2017; Zand et al. 2017) were used. The most important criteria used to decide which papers are suitable to review was having some reports and direct studies on alien plants in Iran. Expert observations were also considered for the species list.

Overview of the search results - 70 records

Unique Identifier Prefix – SouthAsia_Iran_plants

Fish/South Asia

Assessor(s) – Vidyadhar Atkore

Search language(s) – English

Search terms – “invasive fish”, 'exotic fish', "introduced fish species”, “invasive alien fish species” AND “impacts”, "costs" AND “country”

[For country: “India", “Nepal”, “Bhutan”, “Pakistan", “Bangladesh", "Sri Lanka", “Maldives”, “Iran”, “Afghanistan”]

Search engine – Google Scholar

Date - 07.04.2020 to 10.05.2020

The assessor also searched selected authors' bibliographies by visiting their website or Google Scholar page and relevant literature given in the 'references cited' section in each of the papers when available online.

Overview of the search results - 229 records

Unique Identifier Prefix – VA_fish

Multiple taxa/South Asia

Assessor(s) – Ankila Hiremath

Search language(s) – English

I. Preliminary search:

Search terms – (invasive + alien + species + India) + (impact OR cost OR benefit)

Search engine – Google Scholar

Date – September 2019

II. Birds:

Search terms – ("introduced" OR "invasive" OR "alien" OR "IAS" OR "non-native" OR "non-indigenous" OR "pest" OR "exotic") AND "bird" AND ("impacts" OR "costs") AND "Country"

[NOTE: "Country" = India; Nepal; Bhutan; Bangladesh; Sri Lanka; Maldives; Pakistan; Afghanistan; Iran]

Search engine – Google Scholar

Date – April 2020

III. Animals/mammals:

Search terms – ("introduced" OR "invasive" OR "alien" OR "IAS" OR "non-native" OR "non-indigenous" OR "pest" OR "exotic") AND ("animal" OR "mammal") AND ("impacts" OR "costs") AND "Country"

[NOTE: "Country" = India; Nepal; Bhutan; Bangladesh; Sri Lanka; Maldives; Pakistan; Afghanistan; Iran]

Search engine – Google Scholar

Date – 8 April 2020

IV. Reptiles, Amphibians:

Search terms – ("introduced" OR "invasive" OR "alien" OR "IAS" OR "non-native" OR "non-indigenous" OR "pest" OR "exotic") AND ("reptile" OR "amphibian" OR "anuran") AND ("impacts" OR "costs") AND "Country"

[NOTE: "Country" = India; Nepal; Bhutan; Bangladesh; Sri Lanka; Maldives; Pakistan; Afghanistan; Iran]

Search engine – Google Scholar

Date – April 2020

V. Pests, insects, parasites, pathogens:

Search terms – ("introduced" OR "invasive" OR "alien" OR "IAS" OR "non-native" OR "non-indigenous" OR "exotic") AND ("pest" OR "parasite" OR "pathogen" OR "insect") AND ("impacts" OR "costs") AND "India"

[NOTE: "Country" = India; Nepal; Bhutan; Bangladesh; Sri Lanka; Maldives; Pakistan; Afghanistan; Iran]

Search engine – Google Scholar

Date – April 2020

VI. Plants:

Search terms – ("introduced" OR "invasive" OR "alien" OR "IAS" OR "non-native" OR "non-indigenous" OR "exotic") AND ("plant" OR "weed") AND ("impacts" OR "costs") AND "[country]"

[NOTE: "Country" = India; Nepal; Bhutan; Bangladesh; Sri Lanka; Maldives; Pakistan; Afghanistan. Iran was not included, as a CA was conducting a search on Plants/Iran.]

Search engine – Google Scholar

Date – January to March 2021

Article titles, abstracts were checked to make sure that articles included impacts of invasive species. Articles reporting on invasive species distributions, or just comprising an inventory of invasive species were omitted. Articles cited in review papers were also searched and reviewed.

Overview of the search results - 616 records

Unique Identifier Prefix – SouthAsiaMultiple, South_Asia_Multiple

Plants, microbes, invertebrates/Oceania

Assessor(s) – Ellen Ryan-Colton, Eckehard Brockerhoff, Helen Nahrung

Search language(s) – English

Search terms –

II. Australia and New Zealand only: (exotic OR invasi* OR weed) AND (plant*) AND (impact OR effect) AND (Australia OR "New Zealand") refined by type of article: review

II. (exotic OR invasi* OR weed) AND (plant*) AND (impact OR effect) AND (Australia OR "New Zealand") AND (biodiversity)

III. For more information on NCP and GQL impact: (exotic OR invasi* OR weed) AND (plant*) AND (impact OR effect) AND (Australia OR "New Zealand") AND (biodiversity)AND (cost OR benefit OR negative OR positive OR economic OR social OR cultural OR *being)

IV. Search expanded to Oceania: impact* AND invasi* AND polynesia AND (culture OR economic OR ecosystem AND services OR soci* OR *being OR livelihood)

V. impact* AND invasi* AND "pacific islands" AND (culture OR economic OR people OR communit* OR soci* OR *being) AND NOT marine AND NOT ocean AND NOT fish

VI. New Zealand invertebrates and microbes: (exotic OR invasi* OR pest) AND (invertebrate* OR insect*) AND (impact OR effect) AND ("New Zealand") AND (biodiversity OR ecosystem)

(invertebrate AND impact* AND invasi* AND "New Zealand" AND (culture OR economic OR people OR communit* OR soci* OR *being) AND NOT marine AND NOT ocean AND NOT fish

(invasive invertebrate OR alien invertebrate OR non-native invertebrate OR exotic invertebrate OR pest invertebrate) AND (impact OR effect) AND (people) AND (New Zealand)

VII. Australia Forest Pathogens: References contained within Nahrung and Carnegie (2020) for the non-native insects that were scored as having moderate to high impact in forestry (mostly exotic trees, plus timber).

VIII. We also screened data from Oceania from the meta-analysis in Pysek et al 2012: A Global assessment of invasive plant impacts on resident species, communities and ecosystems: the interaction of impact measures, invading species' traits and environment. *Global Change Biology* 18(5). p 1725. and

IX. We also screened data from Oceania from the meta-analysis in Castro-Díez, P. et al. 2019. Global effects of non-native tree species on multiple ecosystem services, *Biological Reviews* 94: 1477-1501. p. brv.12511. doi: 10.1111/brv.12511.

Search engine – Web of Science, Scopus

Date - September 2019 - April 2021

Assessors also searched the references contained within literature sources

Overview of the search results - 710 records

Unique Identifier Prefix –AUSNZPACIFIC, Asia_Pacific_plant_microbes

Ants/Global

Assessor(s) – Ellen Ryan-Colton, working from the database supplied by Gruber et al (2022), authored by Monica Gruber, Davide Santoro, Meghan Cooling, Philip J. Lester, Benjamin D. Hoffmann, Christina Boser, and Lori Lach.

Search language(s) – English

Search terms – Dataset from the global assessment of socio-economic and environmental impacts of invasive ants (Gruber et al 2022), with literature review methods described in Gruber et al (2022). Steps to convert the Gruber et al (2022) dataset to our database with our defined variables were completed for both EICAT (environmental impacts) and SEICAT (socio-economic impacts): 1) Excluded ant species that were not considered alien/exotic were excluded from our analysis, which were defined as ants not recorded as introduced outside their native range, sourced from Gruber et al (2022) using Antweb and Antmaps; 2) Excluded records with impact level of 'Data deficient'; 3) Impact level of MC - minimal concern - assigned as neutral; 4) All other impact records assigned negative direction as positive impacts were not recorded by Gruber et al (2022); 5) accepted all records regardless of confidence rationales; 6) Accepted all sources including online sources (e.g. newspaper articles), which were acceptable as sources by IPBES; 7) Excluded laboratory studies; 9) Assigned Realm as Terrestrial, except for impacts on frogs and fish kills, which were assigned Freshwater, or if Realm was stated elsewhere in text; 10) Assigned other variables, such as Language, Unit of Analysis, Island and so on based the study's location; 11) used EICAT level for Magnitude; 11) Assigned EICAT mechanisms to the standardised categories (e.g. aggression = competition); 12) Assigned NCP or GQL impacts according to text excerpt of each record.

Search engine – Gruber et al (2022) used Web of Science, JSTOR, Google Scholar, and FORMIS, a curated database of all ant literature.

Date - Gruber et al (2022) searched for records prior to March 2019.

Overview of the search results - 844 records

Unique Identifier Prefix – ants

Plants/Caribbean

Assessor(s) – Romina Fernandez, Julissa Sandoval, Natalia Joelson

Search language(s) – English, Spanish

Search terms (plant invader OR exotic plant OR alien plant OR plant inva*) AND (impact OR effect OR change) AND (community OR diversity OR ecosystem process OR ecosystem services OR competition OR allelopathy) AND (Central América OR Caribe OR Belice OR Costa Rica OR El Salvador OR Guatemala OR Honduras OR Nicaragua OR Paraná OR Antigua y Barbuda OR Aruba OR Bahamas OR Barbados OR Cuba OR Dominica OR Granada OR Guadalupe OR Haití OR Jamaica OR Martinica OR Puerto Rico OR República Dominicana OR Santa Lucia OR San Cristobal y Nieves OR San Vicente y las Granadinas OR Trinidad y Tobago)

The assessors also searched the references contained in the sources obtained from the literature search.

Search engine – GoogleScholar; WoS

Date – February-April 2020

Overview of the search results – 131 reports

Unique Identifier Prefix – Carribean_plant

Plants/North America

Assessor(s) – Martin Nunez

Search language(s) – All

Search terms (plant invader OR exotic plant OR alien plant OR plant invasion*) AND (impact* OR effect*) AND (community structure* OR diversity* OR ecosystem process* OR competition*)) AND (USA OR United States OR US OR Canada OR Alabama OR Alaska OR Arizona OR Arkansas OR California OR Colorado OR Connecticut OR Delaware OR Florida OR Georgia OR Idaho OR Illinois OR Indiana OR Iowa OR Kansas OR Kentucky OR Louisiana OR Maine OR Maryland OR Massachusetts OR Michigan OR Minnesota OR Mississippi OR Missouri OR Montana OR Nebraska OR Nevada OR "New Hampshire" OR "New Jersey" OR "New Mexico "OR "New York" OR "North Carolina" OR "North Dakota" OR Ohio OR Oklahoma OR Oregon OR Pennsylvania OR "Rhode Island" OR "South Carolina" OR "South Dakota" OR Tennessee OR Texas OR Utah OR Vermont OR Virginia OR Washington OR "West Virginia" OR Wisconsin OR Wyoming)

Search engine – ISI web of science

Date – April 2020

Overview of the search results – 1932 papers

Unique Identifier Prefix – NA_plant

Vertebrates/Galapagos

Assessor(s) – Diego F. Cisneros-Heredia, Carlos Pazmiño, Paúl Yépez, Henry Rojas

Search language(s) – English, Spanish

Search terms (invasive OR alien OR non-native OR nonnative OR exotic OR ecological invasión OR biological invasión OR invasión biology OR invasión ecology OR invasive species OR introduced species OR nonindigenous OR allochthonous OR exotic) AND (Aves OR Mammalia OR Amphibia OR Reptilia OR bird OR ave OR pájaro OR mamífero OR anfibio OR reptil) AND (Galapagos)

Search terms are provided for Scopus. In Scielo, the search terms were "Galapagos AND (term)," with "term" replaced by previously described keywords. In Google Scholar, the search terms were "Galapagos" "taxon" "term" replaced by previously described keywords.

Search engine – Scopus; GoogleScholar; Scielo

Date – April 2021

Overview of the search results – 211 reports

Unique Identifier Prefix – Galapagos_Vertebrates

Invertebrates/Galapagos

Assessor(s) – Diego F. Cisneros-Heredia, Melannie Nuñez, Milena Campaña, Cristhian Andrade, Paola Espinosa

Search language(s) – English, Spanish

Search terms (invasive OR alien OR non-native OR nonnative OR exotic OR ecological invasión OR biological invasión OR invasión biology OR invasión ecology OR invasive species OR introduced species OR nonindigenous OR allochthonous OR exotic) AND (Hymenoptera OR Hemiptera OR Coleoptera OR Diptera OR Araneae OR fly OR bee OR wasp OR ant OR chinche OR abeja OR avispa OR bug OR beetle OR escarabajo OR mariquita OR gorgojo OR spider OR araña) AND (Galapagos)

Search terms are provided for Scopus. In Scielo, the search terms were "Galapagos AND (term)," with "term" replaced by previously described keywords. In Google Scholar, the search terms were "Galapagos" "taxon" "term" replaced by previously described keywords.

Search engine – Scopus; GoogleScholar; Scielo

Date – April 2021

Overview of the search results – 215 reports

Unique Identifier Prefix – Galapagos_Invertebrates

Vertebrates/Asia Pacific

Assessor(s) – Phill Cassey, Ned L. Ryan-Schofield

Search language(s) – English

Search terms

Search 1

TITLE-ABS-KEY (("introduced species" OR "invasive species" OR "invasive alien species" OR "IAS" OR "alien" OR "non-native" OR "non-indigenous" OR "pest" OR feral" OR "exotic") AND ("Aus*" OR "Oceania") AND ("impact*" OR "effect*" OR "affect*") AND ("vertebra*"))

Search 2

TITLE-ABS-KEY (("introduced species" OR "invasive species" OR "invasive alien species" OR "IAS" OR "alien" OR "non-native" OR "non-indigenous" OR "pest" OR "feral" OR "exotic") AND ("Aus*" OR "Oceania") AND ("impacts" OR "impact" OR "effect" OR "effects" OR "influence" OR "influences") AND ("Bufo marinus" OR "Rhinella marina" OR "cane toad*")) 196 results

Search 3

TITLE-ABS-KEY (("introduced species" OR "invasive species" OR "invasive alien species" OR "IAS" OR "alien" OR "non-native" OR "non-indigenous" OR "pest" OR "feral" OR "exotic") AND ("Aus*" OR "Oceania") AND ("impacts" OR "impact" OR "effect" OR "effects" OR "influence" OR "influences") AND ("camel*" OR "camelus")) 60 results

Search 5

TITLE-ABS-KEY (("introduced species" OR "invasive species" OR "invasive alien species" OR "IAS" OR "alien" OR "non-native" OR "non-indigenous" OR "pest" OR "feral" OR "exotic") AND ("Aus*" OR "Oceania") AND ("donkey*" OR "equus asinus"))

Search 6

TITLE-ABS-KEY (("introduced species" OR "invasive species" OR "invasive alien species" OR "IAS" OR "alien" OR "non-native" OR "non-indigenous" OR "pest" OR "feral" OR "exotic") AND ("Aus*" OR "Oceania") AND ("buffalo*" OR "Bubalus bubalis")) 106 results

Search 7

TITLE-ABS-KEY (("introduced species" OR "invasive species" OR "invasive alien species" OR "IAS" OR "alien" OR "non-native" OR "non-indigenous" OR "pest" OR "feral" OR "exotic") AND ("Aus*" OR "Oceania" OR "New Zealand" OR "American Samoa" OR "Cook Islands" OR "Fiji" OR "French Polynesia" OR "Guam" OR "Kiribati" OR "Marshall Islands" OR "Federated states

of Micronesia" OR "Nauru" OR "New Caledonia" OR "Niue" OR "Northern Mariana Islands" OR "Palau" OR "Papua New Guinea" OR "Pitcairn Islands" OR "Samoa" OR "Solomon Islands" OR "Tonga") AND ("Sus scrofa" OR "pig*") AND ("impact*" OR "effect*" OR "affect*" OR "influence*")) 295 results

Search 8

TITLE-ABS-KEY (("introduced species" OR "invasive species" OR "invasive alien species" OR "IAS" OR "alien" OR "non-native" OR "non-indigenous" OR "pest" OR "feral" OR "exotic") AND ("Aus*" OR "Oceania" OR "New Zealand" OR "Cook Islands" OR "Fiji" OR "French Polynesia" OR "Kiribati" OR "New Caledonia" OR "Norfolk Island*" OR "Northern Mariana Islands" OR "Pitcairn Islands" OR "melanesia" OR "micronesia" OR "polynesia") AND ("Capra hircus" OR "goat*") AND ("impact*" OR "effect*" OR "affect*" OR "influence*")) 111 results

Search 9

TITLE-ABS-KEY(("introduced species" OR "invasive species" OR "invasive alien species" OR "IAS" OR "alien" OR "non-native" OR "non-indigenous" OR "pest" OR "feral" OR "exotic") AND ("Aus*" OR "Oceania" OR "Fiji" OR "French Polynesia" OR "Guam" OR "New Caledonia" OR "New Zealand" OR "melanesia" OR "micronesia" OR "polynesia") AND ("impacts" OR "impact" OR "effect" OR "effects" OR "influence" OR "influences") AND ("Cyprinus carpio" OR "carp")) 67 results

Search 10 – fish

TITLE-ABS-KEY (("introduced species" OR "invasive species" OR "invasive alien species" OR "IAS" OR "alien" OR "non-native" OR "non- indigenous" OR "pest" OR "feral" OR "exotic") AND ("Australia" OR "New Zealand" OR "Oceania" OR "Australasia" OR "micronesia" OR "polynesia" OR "American Samoa" OR "Cook Islands" OR "Christmas Island" OR "Fiji" OR "French Polynesia" OR "Guam" OR "Kiribati" OR "Marshall Islands" OR "Federated states of Micronesia" OR "Nauru" OR "New Caledonia" OR "Norfolk Island" OR "Northern Mariana Islands" OR "Palau" OR "Papua New Guinea" OR "Pitcairn Islands" OR "Samoa" OR "Solomon Islands" OR "Tonga" OR "Tokelau" OR "Tuvalu" OR "Wallis & Futana" OR "vanuatu") AND ("freshwater fish*") AND ("impact*" OR "influence*" OR "effect*" OR "affect*")) 91 results

Search 11

TITLE-ABS-KEY (("introduced species" OR "invasive species" OR "invasive alien species" OR "IAS" OR "alien" OR "non-native" OR "non-indigenous" OR "pest" OR "feral" OR "exotic") AND ("Australia" OR "New Zealand" OR "Oceania" OR "Australasia" OR "micronesia" OR "polynesia" OR "American Samoa" OR "Cook Islands" OR "Christmas Island" OR "Fiji" OR "French Polynesia" OR "Guam" OR "Kiribati" OR "Marshall Islands" OR "Federated states of Micronesia" OR "Nauru" OR "New Caledonia" OR "Norfolk Island" OR "Northern Mariana Islands" OR "Palau" OR "Papua New Guinea" OR "Pitcairn Islands" OR "Samoa" OR "Solomon Islands" OR "Tonga" OR "Tokelau" OR "Tuvalu" OR "Wallis & Futana" OR "vanuatu") AND ("impact*" OR "influence*" OR "effect*" OR "affect*") AND ("Passer domesticus" OR "sparrow*")) 24 results

S12

TITLE-ABS-KEY (("introduced species" OR "invasive species" OR "invasive alien species" OR "IAS" OR "alien" OR "non-native" OR "non-indigenous" OR "pest" OR "feral" OR "exotic") AND ("Australia" OR "New Zealand" OR "Oceania" OR "Australasia" OR "micronesia" OR "polynesia" OR "American Samoa" OR "Cook Islands" OR "Christmas Island" OR "Fiji" OR "French Polynesia" OR "Guam" OR "Kiribati" OR "Marshall Islands" OR "Federated states of Micronesia" OR "Nauru"

OR "New Caledonia" OR "Norfolk Island" OR "Northern Mariana Islands" OR "Palau" OR "Papua New Guinea" OR "Pitcairn Islands" OR "Samoa" OR "Solomon Islands" OR "Tonga" OR "Tokelau" OR "Tuvalu" OR "Wallis & Futana" OR "vanuatu") AND ("impact*" OR "influence*" OR "effect*" OR "affect*") AND ("Acridotheres" OR "mynah" OR "myna")) 26 results

S13

TITLE-ABS-KEY (("introduced species" OR "invasive species" OR "invasive alien species" OR "IAS" OR "alien" OR "non-native" OR "non-indigenous" OR "pest" OR "feral" OR "exotic") AND ("Australia" OR "New Zealand" OR "Oceania" OR "Australasia" OR "micronesia" OR "polynesia" OR "American Samoa" OR "Cook Islands" OR "Christmas Island" OR "Fiji" OR "French Polynesia" OR "Guam" OR "Kiribati" OR "Marshall Islands" OR "federated states off micronesia" OR "Nauru" OR "New Caledonia" OR "Norfolk Island" OR "Northern Mariana Islands" OR "Palau" OR "Papua New Guinea" OR "Pitcairn Islands" OR "Samoa" OR "Solomon Islands" OR "Tonga" OR "Tokelau" OR "Tuvalu" OR "wallis & futuna" OR "vanuatu") AND ("impact*" OR "influence*" OR "effect*" OR "affect*") AND ("Columba livia" OR "Columba domestica" OR "rock pigeon*" OR "rock dove*")) 3 results

S14

TITLE-ABS-KEY (("introduced species" OR "invasive species" OR "invasive alien species" OR "IAS" OR "alien" OR "non-native" OR "non-indigenous" OR "pest" OR "feral" OR "exotic") AND ("Australia" OR "New Zealand" OR "Oceania" OR "Australasia" OR "micronesia" OR "polynesia" OR "American Samoa" OR "Cook Islands" OR "Christmas Island" OR "Fiji" OR "French Polynesia" OR "Guam" OR "Kiribati" OR "Marshall Islands" OR "federated states off micronesia" OR "Nauru" OR "New Caledonia" OR "Norfolk Island" OR "Northern Mariana Islands" OR "Palau" OR "Papua New Guinea" OR "Pitcairn Islands" OR "Samoa" OR "Solomon Islands" OR "Tonga" OR "Tokelau" OR "Tuvalu" OR "wallis & futuna" OR "vanuatu") AND ("impact*" OR "influence*" OR "effect*" OR "affect*") AND ("mallard" OR "Anas platyrhynchos" OR "Anas oustaleti" OR "Anas boschas")) 6 results

S15

TITLE-ABS-KEY (("introduced species" OR "invasive species" OR "invasive alien species" OR "IAS" OR "alien" OR "non-native" OR "non-indigenous" OR "pest" OR "feral" OR "exotic") AND ("Australia" OR "New Zealand" OR "Oceania" OR "Australasia" OR "micronesia" OR "polynesia" OR "American Samoa" OR "Cook Islands" OR "Christmas Island" OR "Fiji" OR "French Polynesia" OR "Guam" OR "Kiribati" OR "Marshall Islands" OR "federated states off micronesia" OR "Nauru" OR "New Caledonia" OR "Norfolk Island" OR "Northern Mariana Islands" OR "Palau" OR "Papua New Guinea" OR "Pitcairn Islands" OR "Samoa" OR "Solomon Islands" OR "Tonga" OR "Tokelau" OR "Tuvalu" OR "wallis & futuna" OR "vanuatu") AND ("impact*" OR "influence*" OR "effect*" OR "affect*") AND ("Pycnonotus" OR "bulbul")) 11 results

S16

TITLE-ABS-KEY (("introduced species" OR "invasive species" OR "invasive alien species" OR "IAS" OR "alien" OR "non-native" OR "non-indigenous" OR "pest" OR "feral" OR "exotic") AND ("Australia" OR "New Zealand" OR "Oceania" OR "Australasia" OR "micronesia" OR "polynesia" OR "American Samoa" OR "Cook Islands" OR "Christmas Island" OR "Fiji" OR "French Polynesia" OR "Guam" OR "Kiribati" OR "Marshall Islands" OR "federated states off micronesia" OR "Nauru" OR "New Caledonia" OR "Norfolk Island" OR "Northern Mariana Islands" OR "Palau" OR "Papua New Guinea" OR "Pitcairn Islands" OR "Samoa" OR "Solomon Islands" OR "Tonga"

OR "Tokelau" OR "Tuvalu" OR "wallis & futuna" OR "vanuatu") AND ("impact*" OR "influence*" OR "effect*" OR "affect*") AND ("Sturnus vulgaris" OR "starling")) 32 documents

S17

TITLE-ABS-KEY (("introduced species" OR "invasive species" OR "invasive alien species" OR "IAS" OR "alien" OR "non-native" OR "non-indigenous" OR "pest" OR "feral" OR "exotic") AND ("Australia" OR "New Zealand" OR "Oceania" OR "Australasia" OR "micronesia" OR "polynesia" OR "American Samoa" OR "Cook Islands" OR "Christmas Island" OR "Fiji" OR "French Polynesia" OR "Guam" OR "Kiribati" OR "Marshall Islands" OR "federated states off micronesia" OR "Nauru" OR "New Caledonia" OR "Norfolk Island" OR "Northern Mariana Islands" OR "Palau" OR "Papua New Guinea" OR "Pitcairn Islands" OR "Samoa" OR "Solomon Islands" OR "Tonga" OR "Tokelau" OR "Tuvalu" OR "wallis & futuna" OR "vanuatu") AND ("impact*" OR "influence*" OR "effect*" OR "affect*") AND ("Gallus gallus" OR "Gallus domesticus" OR "feral chicken")) 23 results (none relevant to chickens)

S18

TITLE-ABS-KEY (("introduced species" OR "invasive species" OR "invasive alien species" OR "IAS" OR "alien" OR "non-native" OR "non-indigenous" OR "pest" OR "feral" OR "exotic") AND ("Australia" OR "New Zealand" OR "Oceania" OR "Australasia" OR "micronesia" OR "polynesia" OR "American Samoa" OR "Cook Islands" OR "Christmas Island" OR "Fiji" OR "French Polynesia" OR "Guam" OR "Kiribati" OR "Marshall Islands" OR "federated states off micronesia" OR "Nauru" OR "New Caledonia" OR "Norfolk Island" OR "Northern Mariana Islands" OR "Palau" OR "Papua New Guinea" OR "Pitcairn Islands" OR "Samoa" OR "Solomon Islands" OR "Tonga" OR "Tokelau" OR "Tuvalu" OR "wallis & futuna" OR "vanuatu") AND ("impact*" OR "influence*" OR "effect*" OR "affect*") AND (" Branta canadensis" OR "Canada geese"))

S19

TITLE-ABS-KEY (("introduced species" OR "invasive species" OR "invasive alien species" OR "IAS" OR "alien" OR "non-native" OR "non-indigenous" OR "pest" OR "feral" OR "exotic") AND ("Australia" OR "New Zealand" OR "Oceania" OR "Australasia" OR "micronesia" OR "polynesia" OR "American Samoa" OR "Cook Islands" OR "Christmas Island" OR "Fiji" OR "French Polynesia" OR "Guam" OR "Kiribati" OR "Marshall Islands" OR "federated states off micronesia" OR "Nauru" OR "New Caledonia" OR "Norfolk Island" OR "Northern Mariana Islands" OR "Palau" OR "Papua New Guinea" OR "Pitcairn Islands" OR "Samoa" OR "Solomon Islands" OR "Tonga" OR "Tokelau" OR "Tuvalu" OR "wallis & futuna" OR "vanuatu") AND ("impact*" OR "influence*" OR "effect*" OR "affect*") AND ("Estrilda astrild" OR "Common waxbill")) 1 result

None adhoc google scholar searching

S20

TITLE-ABS-KEY (("introduced species" OR "invasive species" OR "invasive alien species" OR "IAS" OR "alien" OR "non-native" OR "non-indigenous" OR "pest" OR "feral" OR "exotic") AND ("Australia" OR "New Zealand" OR "Oceania" OR "Australasia" OR "micronesia" OR "polynesia" OR "American Samoa" OR "Cook Islands" OR "Christmas Island" OR "Fiji" OR "French Polynesia" OR "Guam" OR "Kiribati" OR "Marshall Islands" OR "federated states off micronesia" OR "Nauru" OR "New Caledonia" OR "Norfolk Island" OR "Northern Mariana Islands" OR "Palau" OR "Papua New Guinea" OR "Pitcairn Islands" OR "Samoa" OR "Solomon Islands" OR "Tonga" OR "Tokelau" OR "Tuvalu" OR "wallis & futuna" OR "vanuatu") AND ("impact*" OR

"influence*" OR "effect*" OR "affect*") AND ("Bubulcus ibis" OR "Ardea ibis" OR "Ardeola ibis" OR "cattle egret")) 1 result not relevant

S21

TITLE-ABS-KEY (("introduced species" OR "invasive species" OR "invasive alien species" OR "IAS" OR "alien" OR "non-native" OR "non-indigenous" OR "pest" OR "feral" OR "exotic") AND ("Australia" OR "New Zealand" OR "Oceania" OR "Australasia" OR "micronesia" OR "polynesia" OR "American Samoa" OR "Cook Islands" OR "Christmas Island" OR "Fiji" OR "French Polynesia" OR "Guam" OR "Kiribati" OR "Marshall Islands" OR "federated states off micronesia" OR "Nauru" OR "New Caledonia" OR "Norfolk Island" OR "Northern Mariana Islands" OR "Palau" OR "Papua New Guinea" OR "Pitcairn Islands" OR "Samoa" OR "Solomon Islands" OR "Tonga" OR "Tokelau" OR "Tuvalu" OR "wallis & futuna" OR "vanuatu") AND ("impact*" OR "influence*" OR "effect*" OR "affect*") AND ("Corvus splendens")) 1 result not relevant no ad-hoc results

S22

TITLE-ABS-KEY (("new zealand") AND ("impact*" OR "influence*" OR "effect*" OR "affect*") AND ("Alectoris chukar" OR "chukar partridge" OR "Gymnorhina tibicen" OR "magpie*")) 19 results

S23 - deer

TITLE-ABS-KEY (("introduced species" OR "invasive species" OR "invasive alien species" OR "IAS" OR "alien" OR "non-native" OR "non-indigenous" OR "pest" OR "feral" OR "exotic") AND ("Australia" OR "New Zealand" OR "Oceania" OR "Australasia" OR "micronesia" OR "polynesia" OR "American Samoa" OR "Cook Islands" OR "Christmas Island" OR "Fiji" OR "French Polynesia" OR "Guam" OR "Kiribati" OR "Marshall Islands" OR "federated states off micronesia" OR "Nauru" OR "New Caledonia" OR "Norfolk Island" OR "Northern Mariana Islands" OR "Palau" OR "Papua New Guinea" OR "Pitcairn Islands" OR "Samoa" OR "Solomon Islands" OR "Tonga" OR "Tokelau" OR "Tuvalu" OR "wallis & futuna" OR "vanuatu") AND ("impact*" OR "influence*" OR "effect*" OR "affect*") AND ("deer")) 100 results

S24 – possums

TITLE-ABS-KEY (("introduced species" OR "invasive species" OR "invasive alien species" OR "IAS" OR "alien" OR "non-native" OR "non-indigenous" OR "pest" OR "feral" OR "exotic") AND ("New Zealand") AND ("impact*" OR "influence*" OR "effect*" OR "affect*") AND ("Trichosurus vulpecula" OR "brushtail possum")) 187 results

S25 – rats

Results from references for your query: TITLE-ABS-KEY (("introduced species" OR "invasive species" OR "invasive alien species" OR "IAS" OR "alien" OR "non-native" OR "non-indigenous" OR "pest" OR "feral" OR "exotic") AND ("Australia" OR "New Zealand" OR "Oceania" OR "Australasia" OR "micronesia" OR "polynesia" OR "American Samoa" OR "Cook Islands" OR "Christmas Island" OR "Fiji" OR "French Polynesia" OR "Guam" OR "Kiribati" OR "Marshall Islands" OR "federated states off micronesia" OR "Nauru" OR "New Caledonia" OR "Norfolk Island" OR "Northern Mariana Islands" OR "Palau" OR "Papua New Guinea" OR "Pitcairn Islands" OR "Samoa" OR "Solomon Islands" OR "Tonga" OR "Tokelau" OR "Tuvalu" OR "wallis & futuna" OR "vanuatu") AND ("impact*" OR "influence*" OR "effect*" OR "affect*") AND ("rattus

rattus" OR "ship rat" OR "black rat" OR "rattus norvegicus" OR "brown rat" OR "rattus exulans" OR "pacific rat")) 177 results

S25 – rabbits

TITLE-ABS-KEY (("introduced species" OR "invasive species" OR "invasive alien species" OR "IAS" OR "alien" OR "non-native" OR "non-indigenous" OR "pest" OR "feral" OR "exotic") AND ("Australia" OR "New Zealand" OR "New Caledonia" OR "Kiribati" OR "French Polynesia" OR "Norfolk Island") AND ("impact*" OR "influence*" OR "effect*" OR "affect*") AND ("Oryctolagus cuniculus" OR "Lepus cuniculus" OR "European rabbit*")) 238 results

S26 – mice

Results from references for your query: TITLE-ABS-KEY (("introduced species" OR "invasive species" OR "invasive alien species" OR "IAS" OR "alien" OR "non-native" OR "non-indigenous" OR "pest" OR "feral" OR "exotic") AND ("Australia" OR "New Zealand" OR "Oceania" OR "Australasia" OR "micronesia" OR "polynesia" OR "American Samoa" OR "Cook Islands" OR "Christmas Island" OR "Fiji" OR "French Polynesia" OR "Guam" OR "Kiribati" OR "Marshall Islands" OR "federated states off micronesia" OR "Nauru" OR "New Caledonia" OR "Norfolk Island" OR "Northern Mariana Islands" OR "Palau" OR "Papua New Guinea" OR "Pitcairn Islands" OR "Samoa" OR "Solomon Islands" OR "Tonga" OR "Tokelau" OR "Tuvalu" OR "wallis & futuna" OR "vanuatu") AND ("impact*" OR "influence*" OR "effect*" OR "affect*") AND ("Mus musculus" OR “Mus domesticus”)) 129 results

S27 – reptiles

TITLE-ABS-KEY (("introduced species" OR "invasive species" OR "invasive alien species" OR "IAS" OR "alien" OR "non-native" OR "non-indigenous" OR "pest" OR "feral" OR "exotic") AND ("Australia" OR "New Zealand" OR "Oceania" OR "Australasia" OR "micronesia" OR "polynesia" OR "American Samoa" OR "Cook Islands" OR "Christmas Island" OR "Fiji" OR "French Polynesia" OR "Guam" OR "Kiribati" OR "Marshall Islands" OR "federated states off micronesia" OR "Nauru" OR "New Caledonia" OR "Norfolk Island" OR "Northern Mariana Islands" OR "Palau" OR "Papua New Guinea" OR "Pitcairn Islands" OR "Samoa" OR "Solomon Islands" OR "Tonga" OR "Tokelau" OR "Tuvalu" OR "wallis & futuna" OR "vanuatu") AND ("impact*" OR "influence*" OR "effect*" OR "affect*") AND ("Hemidactylus frenatus" OR "common house gecko" OR "Ramphotyphlops braminus" OR "Brahminy blindsnake" OR "Varanus indicus" OR "mangrove monitor" OR "Boiga irregularis" OR "brown tree snake" OR "Trachemys scripta elegans" OR "Red eared slider" OR "Iguana iguana" OR "green iguana" OR "Norops sagrei" OR "Bahamian brown anole" OR "Anolis carolinensis" OR "green anole")) 94 results

S28 – Mustelids

TITLE-ABS-KEY (("introduced species" OR "invasive species" OR "invasive alien species" OR "IAS" OR "alien" OR "non-native" OR "non-indigenous" OR "pest" OR "feral" OR "exotic") AND ("Australia" OR "New Zealand") AND ("impact*" OR "influence*" OR "effect*" OR "affect*") AND ("Mustela")) 105 results

S29 – Foxes

TITLE-ABS-KEY (("introduced species" OR "invasive species" OR "invasive alien species" OR "IAS" OR "alien" OR "non-native" OR "non-indigenous" OR "pest" OR "feral" OR "exotic") AND ("Australia") AND ("impact*" OR "influence*" OR "effect*" OR "affect*") AND ("Vulpes vulpes" OR "red fox")) 189 results

S30 – cats

TITLE-ABS-KEY (("introduced species" OR "invasive species" OR "invasive alien species" OR "IAS" OR "alien" OR "non-native" OR "non-indigenous" OR "pest" OR "feral" OR "exotic") AND ("Australia" OR "New Zealand" OR "Hawaii" OR "Oceania" OR "Australasia" OR "micronesia" OR "polynesia" OR "American Samoa" OR "Cook Islands" OR "Christmas Island" OR "Fiji" OR "French Polynesia" OR "Guam" OR "Kiribati" OR "Marshall Islands" OR "federated states off micronesia" OR "Nauru" OR "New Caledonia" OR "Norfolk Island" OR "Northern Mariana Islands" OR "Palau" OR "Papua New Guinea" OR "Pitcairn Islands" OR "Samoa" OR "Solomon Islands" OR "Tonga" OR "Tokelau" OR "Tuvalu" OR "wallis & futuna" OR "vanuatu") AND ("impact*" OR "influence*" OR "effect*" OR "affect*") AND ("Felis catus" OR "Felis silvestris catus" OR "Felis domesticus" OR "cat")) 388 results

S31 – dogs

TITLE-ABS-KEY (("introduced species" OR "invasive species" OR "invasive alien species" OR "IAS" OR "alien" OR "non-native" OR "non-indigenous" OR "pest" OR "feral" OR "exotic") AND ("Australia" OR "New Zealand" OR "Hawaii" OR "Oceania" OR "Australasia" OR "micronesia" OR "polynesia" OR "American Samoa" OR "Cook Islands" OR "Christmas Island" OR "Fiji" OR "French Polynesia" OR "Guam" OR "Kiribati" OR "Marshall Islands" OR "federated states off micronesia" OR "Nauru" OR "New Caledonia" OR "Norfolk Island" OR "Northern Mariana Islands" OR "Palau" OR "Papua New Guinea" OR "Pitcairn Islands" OR "Samoa" OR "Solomon Islands" OR "Tonga" OR "Tokelau" OR "Tuvalu" OR "wallis & futuna" OR "vanuatu") AND ("impact*" OR "influence*" OR "effect*" OR "affect*") AND ("Canis lupus" OR "Canis familiaris" OR "Canis dingo" OR "dog")) 189 results

Search engine – GoogleScholar; Scopus; Web of Science

Date – April 2020

Overview of the search results – 1052 impacts

Unique Identifier Prefix – Oceania_vertebrates

Literature cited

Capinha, C., Seebens, H., Cassey, P., García-Díaz, P., Lenzner, B., Mang, T., ... & Essl, F. (2017). Diversity, biogeography and the global flows of alien amphibians and reptiles. *Diversity and Distributions*, *23*(11), 1313-1322.

Diagne, Christophe, et al. "High and rising economic costs of biological invasions worldwide." Nature 592.7855 (2021): 571-576.

Duenas, Manuel-Angel, et al. "The role played by invasive species in interactions with endangered and threatened species in the United States: a systematic review." Biodiversity and Conservation 27.12 (2018): 3171-3183.

Evans, T., Kumschick, S., & Blackburn, T. M. (2016). Application of the Environmental Impact Classification for Alien Taxa (EICAT) to a global assessment of alien bird impacts. Diversity and Distributions, 22(9), 919-931.

Evans, T., Blackburn, T. M., Jeschke, J. M., Probert, A. F., & Bacher, S. (2020). Application of the Socio- Economic Impact Classification for Alien Taxa (SEICAT) to a global assessment of alien bird impacts. NeoBiota, 62, 123.

Gallardo, Belinda, et al. "Global ecological impacts of invasive species in aquatic ecosystems." Global change biology 22.1 (2016): 151-163.

Gruber, Monica A. M., Davide Santoro, Meghan Cooling, Philip J. Lester, Benjamin D. Hoffmann, Christina Boser, and Lori Lach. 2022. " A Global Review of Socioeconomic and Environmental Impacts of Ants Reveals New Insights for Risk Assessment." *Ecological Applications* 32(4): e2577. https://doi.org/10.1002/eap.2577

Gutiérrez, J. L., Jones, C. G., & Sousa, R. (2014). Toward an integrated ecosystem perspective of invasive species impacts. *Acta Oecologica*, *54*, 131-138.

Hagen BL, Kumschick S. 2018. The relevance of using various scoring schemes revealed by an impact assessment of feral mammals. NeoBiota 38: 37-75.

https://doi.org/10.3897/neobiota.38.23509

Hanley, Nicholas, and Michaela Roberts. "The economic benefits of invasive species management. "People and Nature 1.2 (2019): 124-137.

Howard, Patricia L. "Human adaptation to invasive species: A conceptual framework based on a case study metasynthesis." Ambio 48.12 (2019): 1401-1430.

Kapitza, Katharina, et al. "Research on the social perception of invasive species: a systematic literature review." NeoBiota 43 (2019): 47.

Marr SM, Ellender BR, Woodford DJ, Alexander ME, Wasserman RJ, Ivey P, Zengeya TA, Wely OLF. 2017. Evaluating invasion risk for freshwater fishes in South Africa. Bothalia 47: a2177. https://doi.org/10.4102/abc.v47i2.2177

Measey, G. J., Vimercati, G., De Villiers, F. A., Mokhatla, M., Davies, S. J., Thorp, C. J., ... & Kumschick, S. (2016). A global assessment of alien amphibian impacts in a formal framework. Diversity and Distributions, 22(9), 970-981.

Nahrung, H.F. and Carnegie, A.J. (2020) Non-native Forest Insects and Pathogens in Australia: Establishment, Spread, and Impact. *Frontiers in Forests and Global Change* 3:37. doi: 10.3389/ffgc.2020.00037

Olson, Lars J. "The economics of terrestrial invasive species: a review of the literature." Agricultural and Resource Economics Review 35.1 (2006): 178-194.

Pimentel, David, Rodolfo Zuniga, and Doug Morrison. "Update on the environmental and economic costs associated with alien-invasive species in the United States." Ecological economics 52.3 (2005): 273-288.

Sanderson, L. A., J. A. McLaughlin, and P. M. Antunes. "The last great forest: a review of the status of invasive species in the North American boreal forest." Forestry 85.3 (2012): 329-340.

Shackleton, Ross T., Charlie M. Shackleton, and Christian A. Kull. "The role of invasive alien species in shaping local livelihoods and human well-being: A review." Journal of environmental management 229 (2019): 145-157.

Shivambu TC, Shivambu N, Downs CT. 2020. Impact assessment of seven alien invasive bird species already introduced to South Africa. Biological Invasions 22: 1829-1847. https://doi.org/10.1007/s10530-020-02221-9

Sohrabi S, Gherekhloo J, Rashed Mohasel MH (2017) Plant invasion and invasive weeds of Iran. Mashhad University Press, Mashhad

Van Wilgen B, Zengeya TA, Richardson D. 2022. A review of the impacts of invasive alien species in South Africa. Biological Invasions 24: 27-50. https://doi.org/10.1007/s10530-021-02623-3

Volery, L., Jatavallabhula, D., Scillitani, L., Bertolino, S., & Bacher, S. (2021). Ranking alien species based on their risks of causing environmental impacts: A global assessment of alien ungulates. Global Change Biology, 27(5), 1003-1016.

Yan, Xie, et al. "Invasive species in China—an overview." Biodiversity & Conservation 10.8 (2001): 1317-1341. JWRC (ed) (2008, 2019) A Photographic Guide to the Invasive Alien Species in Japan. Heibonsha, Tokyo (in Japanese)

Zand E, Baghestani MA, Nezamabadi N, Shimi P, Mousavi SK (2017) A guide to chemical control of weeds in Iran: In regard to weeds shifts. Mashhad University Press, Mashhad
